# Untargeted metabolomics of the intestinal tract of DEV-infected ducks

**DOI:** 10.1186/s12985-023-02266-x

**Published:** 2023-12-19

**Authors:** Haiqing Cai, Xia Yang, Yunyun Yang, Yi Feng, Anlin Wen, Ying Yang, Ming Wen, Deyuan Ou

**Affiliations:** 1 School of Animal Science, Guizhou University, Guiyang, China; 2 Guizhou Provincial Animal Biological Products Engineering Technology Research Center, Guiyang, China

**Keywords:** Duck enteritis virus, Duck, Gut, Differentially abundant metabolites, Nontargeted metabolomics

## Abstract

**Introduction:**

Duck enteritis virus (DEV) mainly causes infectious diseases characterized by intestinal haemorrhage, inflammation and parenchymal organ degeneration in ducks and other poultry. However, the mechanism by which it causes intestinal damage in ducks is not well understood. Metabolomics can provide an in-depth understanding of the full complexity of the disease.

**Methods:**

In this study, 24 clinically healthy green-shell ducks (weight 1.5 kg ± 20 g) were randomly divided into 2 groups (experimental group, 18; control group, 6). The experimental group was intramuscularly injected with 0.2 mL of DEV virus in solution (TCID_50_ 3.16 × 10^8^ PFU/mL), and the control group was injected with 0.2 mL of sterile normal saline. Duck duodenum and ileum tissue samples were collected at 66 h, 90 h and 114 h post-injection (12 h of fasting before killing), and metabolomics analysis of duck duodenum and ileum tissues at the three time points (66, 90, 114 h) was performed by liquid chromatography–mass spectrometry (LC–MS) to screen for and analyse the potential differentiated metabolites and related signalling pathways.

**Results:**

Screening was performed in the positive/negative mode (Pos: Positive ion mode; the ionization of substances at the ion source with positive ions such as H^+^, NH_4_^+^, Na^+^ and K^+^; Neg: Negative ion mode; the ionization of substances at the ion source with negative ions such as Cl^−^, OAc^−^), and compound abundance was compared to that in the control group. The total number of differentially abundant compounds in the duodenum at 66 h, 90 h and 114 h of DEV infection gradually increased, and metabolites such as cytidine, 2′-deoxyriboside and 4-guanidinobutyric acid were differentially abundant metabolites common to all three time periods. The metabolic pathways related to inflammatory response and immune response were tryptophan acid metabolism, cysteine-methionine metabolism, histidine metabolism and other amino acid metabolism and fat metabolism. Among them, the metabolic pathways with more differentially abundant metabolites were amino acid biosynthesis, cysteine and methionine metabolism, tryptophan metabolism, unsaturated fatty acid biosynthesis and purine metabolism, and the metabolic pathways with more enrichment factors were the IgA-related intestinal immune network pathway and lysosome pathway. Compared with the control group, there were 16 differentially abundant metabolites in the ileum tissue of DEV-infected ducks at 66 h of infection, 52 at 90 h of infection, and 40 at 14 h of infection with TD114. The metabolic pathways with more enriched differentially abundant metabolites were pyrimidine metabolism, tyrosine metabolism, phenylalanine metabolism and tryptophan biosynthesis. The metabolic pathways with the most enrichment factors were the mTOR signalling pathway, ferroptosis pathway, tryptophan metabolism pathway and caffeine metabolism pathway.

**Conclusion:**

Comparative analysis showed that the number of differentially abundant metabolites in the duodenum and ileum differed to some extent after DEV infection, with significantly more differentially abundant metabolites in duodenal tissues and fewer in ileal tissues; after DEV infection, the highest number of differentially abundant metabolites was obtained at 114 h of DEV infection, followed by the second highest at 90 h of infection and the lowest at 66 h of infection. The common differentially abundant metabolites in duodenal and ileal tissues were prostaglandins, arachidonic acid, and arachidonic ethanolamine. The main metabolic pathways in the duodenum were the IgA-associated intestinal immune network pathway and the lysosomal pathway, and the metabolic pathways with more enriched factors in the ileum were the mTOR signalling pathway, the ferroptosis pathway, and the tryptophan metabolism pathway.

**Supplementary Information:**

The online version contains supplementary material available at 10.1186/s12985-023-02266-x.

## Introduction

Duck enteritis virus (DEV), also known as duck plague virus (DPV), has a typical herpesvirus structure: the virion is spherical and has a capsule, and its genome consists of 158,091 bp of double-stranded linear DNA [1]. It can cause acute septic and highly contagious infectious disease in ducks, geese, and a variety of Anseridae poultry. DEV is an important pathogen that seriously threatens the development of the duck industry [2]. It mainly replicates in the mucosa of the digestive tract and then spreads to the bursa, thymus, spleen and liver [3].

Untargeted metabolomics enables systematic access to biological compounds metabolized by DEV in ducks, such as amino acids and related amines, lipids, sugars, nucleotides, and other intermediate metabolites [4]. By biological definition, targeted metabolites are known and represent specific pathways or molecular classes in metabolomics, whereas untargeted metabolomics mainly identifies differential metabolites. Untargeted metabolomics, i.e., the discovery of metabolites, can identify differences between metabolite profiles associated with specific biological conditions [5]. Untargeted metabolomics uses LC‒MS, GC‒MS, NMR and other techniques to detect all small molecule metabolites (mainly endogenous small molecule compounds with a relative molecular weight less than 1000 Da) in cells, tissues, organs or organisms without bias, dynamic changes before and after stimulation or interference, screening differentially abundant metabolites through bioinformatics analysis, and conducting pathway analysis on differentially abundant metabolites to reveal the physiological mechanism of their changes [6]. At present, scholars at home and abroad have not stopped research on DEV, but they are all basic research, and the material changes in the development of the pathogenic mechanism of DEV are less involved, especially metabolome sequencing of the host's anti-viral infection response analysis [7]. At present, our laboratory has completed transcriptome sequencing of DEV-infected duck duodenum and high-throughput sequencing of the duodenum, although metabolome sequencing technology has been widely used in drug research and development or different stages of livestock and poultry as well as outside world substance changes under changing conditions. To date, no studies have reported on the changes in intestinal tissue in ducks after DEV infection.

In this study, we used LC–MS technology to detect the dynamic changes in all small molecule metabolites in the intestines caused by DEV infection of ducks, screened differentially abundant metabolites by bioinformatics analysis, and performed pathway analysis of differentially abundant metabolites to understand the mechanisms and changes in intestinal metabolic disorders in ducks after DEV infection.

## Materials and methods

### Virus strains and experimental animals

The Guizhou strain of duck enteritis virus (DEV-GZ strain), provided by Guizhou Provincial Key Laboratory of Animal Epidemiology and Veterinary Public Health, had a TCID_50_ of 3.16 × 10^8^ PFU/mL and was titrated by the Reed and Muench method [8] (Reed—Muench). Twenty-four one-day-old *Anas platyrhynchos* were purchased from Guangdong Mingyan Poultry Industry Co., Ltd., and all were negative for DEV through serological and pathogenic tests. After being boiled and sterilized, they were fed with full-price feed until 41 days of age. All ducks excluded the possibility of several common viruses (i.e., AIV, DHV, NDV) infecting ducks. The experimental operation strictly adhered to the Guidelines for the Breeding and Use of Laboratory Animals [9]. The experimental grouping of TD represents different time periods of DEV-infected groups, CK represents the control group, "duodenum" represents the duodenum, abbreviated as "Du"; "ileum" represents the ileum, abbreviated as "I". All the procedures of sample collection were approved and agreed upon by the Ethics Committee of Guizhou University in accordance with the requirements of animal ethics (approval number EAE-GZU-2021-T005).

### Main reagents and instruments

LC–MS grade acetonitrile (ACN) and methanol (MeOH) were purchased from Fisher Scientific (Loughborough, UK). Formic acid was obtained from TCI (Shanghai, China). Chloroform was obtained from Sinopharm (Shanghai, China). Ultrapure water was generated using a Milli-Q system (0.22 μm, Millipore, Bedford, USA). 2-Amino-3-(2-chloro-phenyl)-propionic acid was obtained from Aladdin (Shanghai, China). Ultrahigh-performance liquid chromatography (UHPLC) and high-resolution mass spectrometry (HRMS) columns were supplied by Shenzhen Huada Genetics Co., Ltd. (Shenzhen, China).

### Establishment of the DEV-infected duck model

Pathological and serological tests in our laboratory were performed in accordance with the requirements of the experiment, and there were no other pathogens present (including DEV, MDV, AIV, etc.). The ducks in the experimental group were inoculated with 0.2 mL of DEV-GZ strain virus each via the leg muscles at 41 days of age, and after the viral challenge, the ducklings were observed for mental status and lesions every 6 h. Blood was collected and analysed at 66 h, 90 h and 114 h. The control group was inoculated with 0.2 mL of sterile saline, and the blood was collected and analysed at 66 h. After 1 mL of blood was collected from the subwing vein, the ducklings were anaesthetized with ether and sacrificed by dislocation of the neck. Tissues such as the duodenum and ileum were collected, and some were stored in a − 80 °C deep freezer for later use. Some were immediately frozen in liquid nitrogen after collection. After all samples from different time periods were obtained, they were sent to Shenzhen BGI Technology Service Co., Ltd. on dry ice for metabolomics sequencing.

### Nucleic acid PCR of intestinal tissues of DEV-infected ducks

Duodenal and ileal samples were collected aseptically from the control and DEV-infected groups and processed by grinding, and then the DNA in the samples was extracted using the Ezup Column DNA Nucleic Acid Extraction Kit for subsequent experiments. The control and DEV-infected groups were used as templates for the detection of the DEV-NP gene using the PCR method established in our laboratory (primers: F 5′-CTGGAAGATGCAGTAACGTCTG-3′, R 5′- CTGGGGTTGTCTGTATTCGGAGT-3′), the viral fluid of the DEV GZ strain was used as a positive control, and ddH_2_O was used as a negative control. The amplified fragment was 133 bp (PCR procedure: predenaturation at 95 °C for 30 s, 95 °C for 5 s, 60 °C for 30 s, 72 °C for 40 s, 40 cycles: extension at 72 °C for 10 min), and the amplified product was detected by electrophoresis on a 1.2% agarose gel.

### Preparation of samples and metabolites

Samples were collected from the control group at 66 h. The ducks were fasted for 12 h before sampling (no water restriction). Three time points were selected for sampling, and duodenum and ileum tissues were collected at each: 66 h, 90 h, and 114 h. The sample size was more than 0.1 g. Six samples in parallel were numbered on the outside of the cryotube with an oily marker pen, according to the number designating one duck. After removing the connective tissue and adipose tissue, the intestinal tissue was rinsed with normal saline, and the sample was clamped with tweezers and placed in a cryotube. The samples were placed in liquid nitrogen overnight and then stored at − 80 °C until they were sent for inspection. TD66-Du, TD90-Du, TD114-Du, TD66-I, TD90-I, TD114-I, CK-Du and CK-I were stored on dry ice and sent to Shenzhen Huada Gene Co., Ltd., and the remaining tissue samples were placed in sterile sampling bags for subsequent analysis of pathogenic nucleic acids and stored at − 80 °C for later use.

### Data processing and quality control

Raw data were exported using Compound Discoverer3.1 software to convert formats for further data analysis. The R software metaX was used to calculate the probability quotient normalization to correct the data of the QC sample, including high noise reduction, peak alignment extraction and normalization, to obtain the relative peak area. The peak in the mixed QC should produce a compound with an RSD of less than 30%. After data normalization using the QC-RLSC method for different batches of tissue, the data were extracted into the data matrix.

Before the experimental sample detection, the stability of the instrument and the balance of the MS system relied on the repeatability of the QC sample detection to evaluate the data quality. Before every 10 samples, the system was injected with blank solvent, mixed quality control samples, and instrument quality control samples of 6 standards. The display of the results includes the chromatogram overlap, PCA, peak number and peak response intensity difference of QC samples.

### Compound test results and notes

The metabolites were obtained using metaX, a metabolomics R software package independently developed by Shenzhen BGI, and identified by online databases such as Chempider, HMDB, and KEGG. MetaboAnalyst4.0 software was used to analyse the biological information of each pathway and network, match the identified compounds, and obtain the main biochemical metabolic pathways and signal transduction pathways in which these metabolites participate.

### Screening of differentially abundant metabolites

The data analysis incorporated VIP and univariate analysis in multivariate statistics at the same time. The *P* value obtained from the t test was used to assess statistical significance. Univariate analysis was used to obtain the FC between groups. Unsupervised principal component analysis (PCA) was performed. The effectiveness of the PLS model was evaluated using the goodness of fit R2. The results of the Q2 test and the goodness of fit of 200 transformation models were compared. The t test was used to screen for potential biomarkers with VIP values exceeding 1 and *P* values lower than 0.05. For the intersection of the criteria FC ≥ 1.2 or FC ≤ 0.83 33, *P* value < 0.05 and VIP ≥ 1, the common ions were considered differential ions Those and marked in red (FC ≥ 1.2 and *P* value < 0.05) or green (FC ≤ 0.8333, and *P* value < 0.05), and the results were visualized using a volcano map.

### Analysis of differentially abundant metabolites

Cluster analysis of differentially abundant metabolites was performed using R software. The pheatmap function uses heatmap (R version 3.5.3) and z score processes, converts Log2 data, calculates Euclidean distance to display the changes in differentially abundant metabolites, and compares the differences between different treatment samples. Simple, intuitive hierarchical clustering was performed. Biological functions were analysed, DEV-related biomarkers were mined, the classification status and function of metabolites were assigned, the database (KEGG) was matched, and literature reports related to inflammation were consulted to determine the biological effects of differentially abundant metabolites in DEV-infected ducks. The main biochemical pathways and signal transduction pathways in which metabolites participate were annotated in the form of pathway diagrams, and then differentially expressed related substances were screened. Biochemical pathway diagrams were drawn for metabolites. Note: N, represents the number of pathways that can be matched in all identified metabolites; n, represents the number of pathways that can be matched in significantly different metabolites; M, represents the number of pathways that can be matched in all identified metabolites; m: represents the number of pathways that can be matched in significantly different metabolites, and the formula for the hypergeometric test of the number of classifications for the differentially expressed substances that are significantly enriched to the pathway is as follows:

$$P = 1 - \sum\limits_{i = 0}^{m - 1} {\frac{{\left( {\begin{array}{*{20}c} M \\ i \\ \end{array} } \right)\left( {\begin{array}{*{20}c} {N - M} \\ {n - i} \\ \end{array} } \right)}}{{\left( {\begin{array}{*{20}c} N \\ n \\ \end{array} } \right)}}}$$

With a *P* value < 0.05 as the threshold, the screened differentially abundant metabolites were significantly enriched in the rntry. The number of pathways related to differentially abundant metabolites pathways and the number of upregulated pathways in this study were counted, and a bubble map of metabolic pathway enrichment analysis was obtained. Metabolic pathways were searched using the web database KEGG (http://www.kegg.jp) to elucidate biological metabolic pathways associated with DEV.

## Results

### Establishment of the DEV infection model

**Fig. 1:**
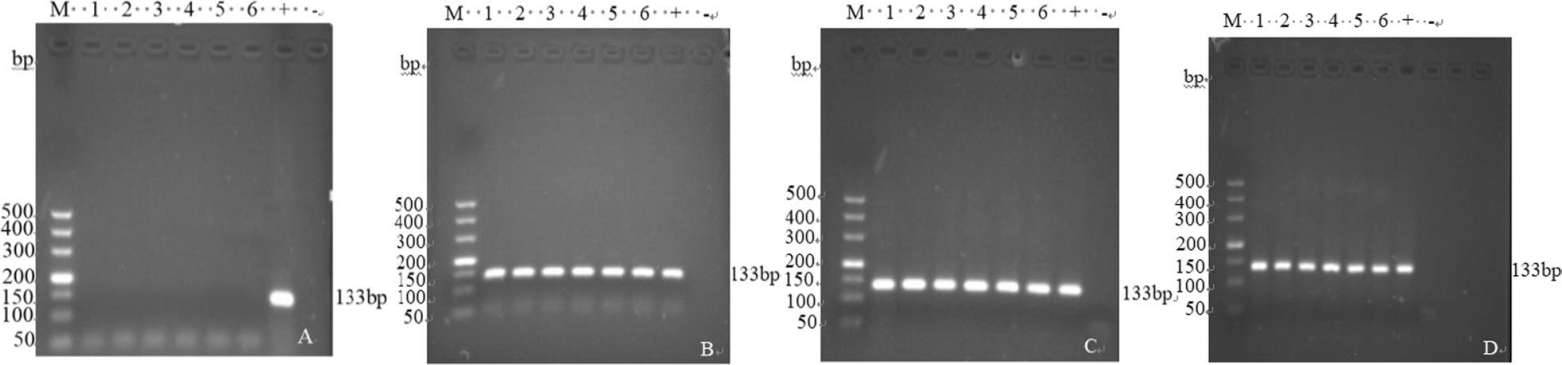
PCR detection results of DEV-NP in intestinal tissues. **A** Control group; **B** DEV 66 h; **C** DEV 90 h; **D** DEV 114 h

DEV detection was carried out on the intestinal sample DNA of ducks in the control group and DEV infection group by PCR. The results (Fig. 1) showed that there was no corresponding band of the DEV-NP gene in the intestinal tissue of ducks in the control group, and corresponding bands could be detected in each period of time in the experimental group. The presence of the band indicated that ducks were infected with DEV, which was used for subsequent screening of differentially abundant metabolites between the control group and the DEV infection group.

### Metabolomics grouping and data processing of ducks infected with DEV

#### Experimental grouping and program design

The control group and the infection group were designed according to the research plan. Since the infection group was divided for sampling at three time points (66 h, 90 h, and 114 h), the grouping information and difference comparison scheme are shown in Table 1. Each group of samples was parallelized 6 times to reduce the variation among samples.

**Table 1:** Group information and scheme

Compare_Group	Compare_Group	Compare_Group
TD66-Du-vs.-CK-Du	TD90-Du-vs.-CK-Du	TD114-Du-vs.-CK-Du
TD66-I-vs.-CK-I	TD90-I-vs.-CK-I	TD114-I-vs.-CK-I

#### Data preprocessing and quality control

After initial preliminary processing of the raw data, the probability quotient-normalized corrected QC samples were calculated using the R software metaX, including high noise reduction, peak alignment extraction and normalization to obtain the relative peak area. The peaks in the mixed QCs yielded compounds with less than 30% RSD, which were extracted into the data matrix after normalizing the data using the QC-RLSC method for different batches of tissues. The results showed that 18,735 compounds were available in positive ion mode, and the proportion of qualified data was 78.98%; 3090 compounds were available in negative ion mode, and the proportion of qualified data was 86.02%. Both percentages were greater than 60%, which suggests that the quality is adequate, reflecting the high amount of data.

1. Chromatograms of QC samples

**Fig. 2:**
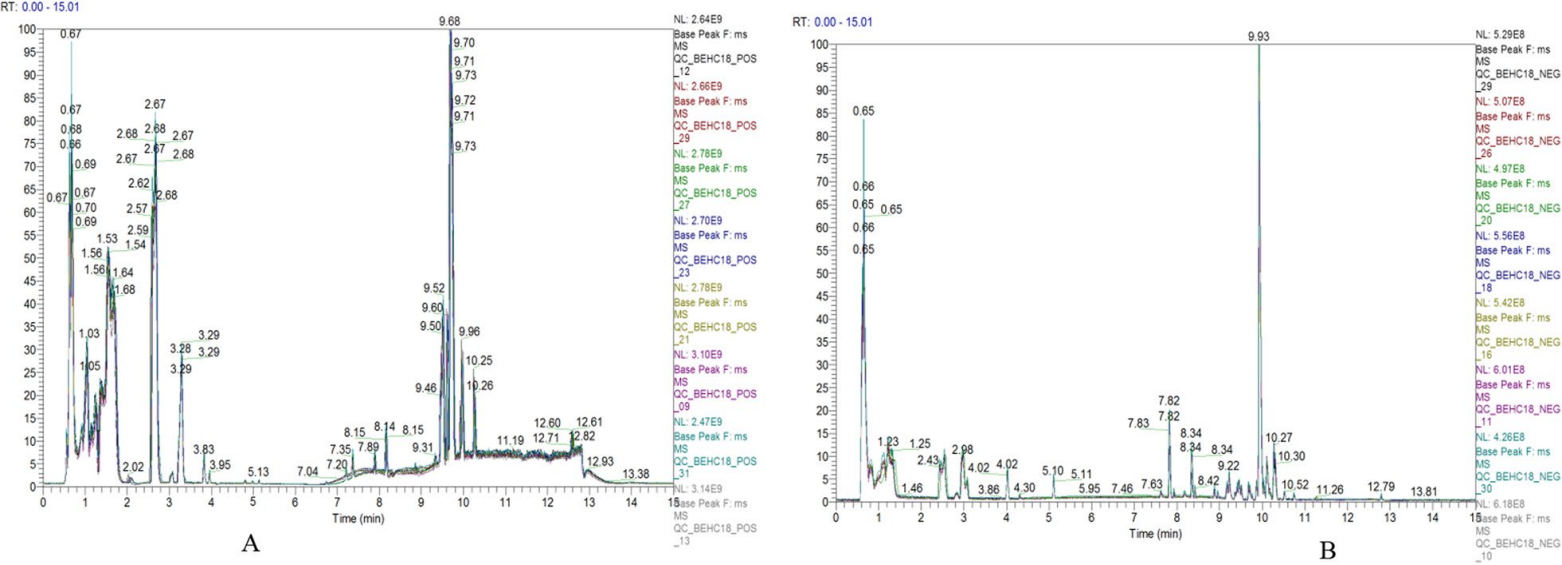
BPC overlay spectrum of QC sample ion mode in two ion modes. **A** positive ion mode; **B** negative ion mode

The BPC spectrum of the sample had a good peak shape and large peak capacity, good spectral overlap, and small fluctuations in retention time and peak response intensity (Fig. 2), indicating that the instrument was in good condition, the signal was stable, the peak shape was consistent, and it only the time when the maximum ion intensity appeared was variable (See Additional file 1 for the remaining subgroup BPC diagrams).

(2) Aggregation analysis of principal components of all samples

**Fig. 3:**
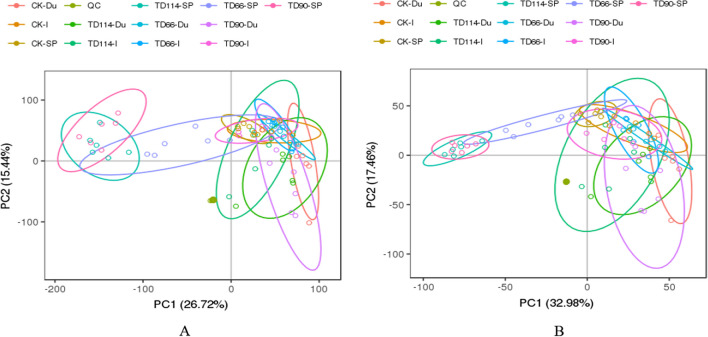
PCA diagram of each tissue sample in positive and negative ion mode. **A** positive ion mode; **B** negative ion mode; the abscissa represents the first principal component, represented by PC1; the ordinate represents the second principal component, represented by PC2

A two-dimensional scatterplot of the PCA model of QC samples (Fig. 3) showed complete separation of the CK and DEV groups, indicating that DEV infection altered the metabolite profiles. In both representative ion modes, there are no obvious outliers, and the separation of samples is relatively good. Although the metabolic profiles partially overlap, the metabolic components are significantly different between the DEV group and the CK group, and the metabolic profiles of TD90-Du, TD66-I, and TD90-I also significantly differed from the metabolic profiles of the other groups.

#### Compound detection results and identification

Multiple databases of the BGI Library, mzCloud and ChemSpider (HMDB, KEGG, LipidMaps) were used to identify compounds, and there was no difference in the results for the same groups in different databases. After identification and quality control of the compounds in the preprocessed data, 14,796 compounds were obtained in positive ion mode, of which 6771 compounds were identified, and 2658 compounds were obtained in negative ion mode, of which 1300 compounds were identified.

#### Compound notes

1. Category Notes

**Fig. 4:**
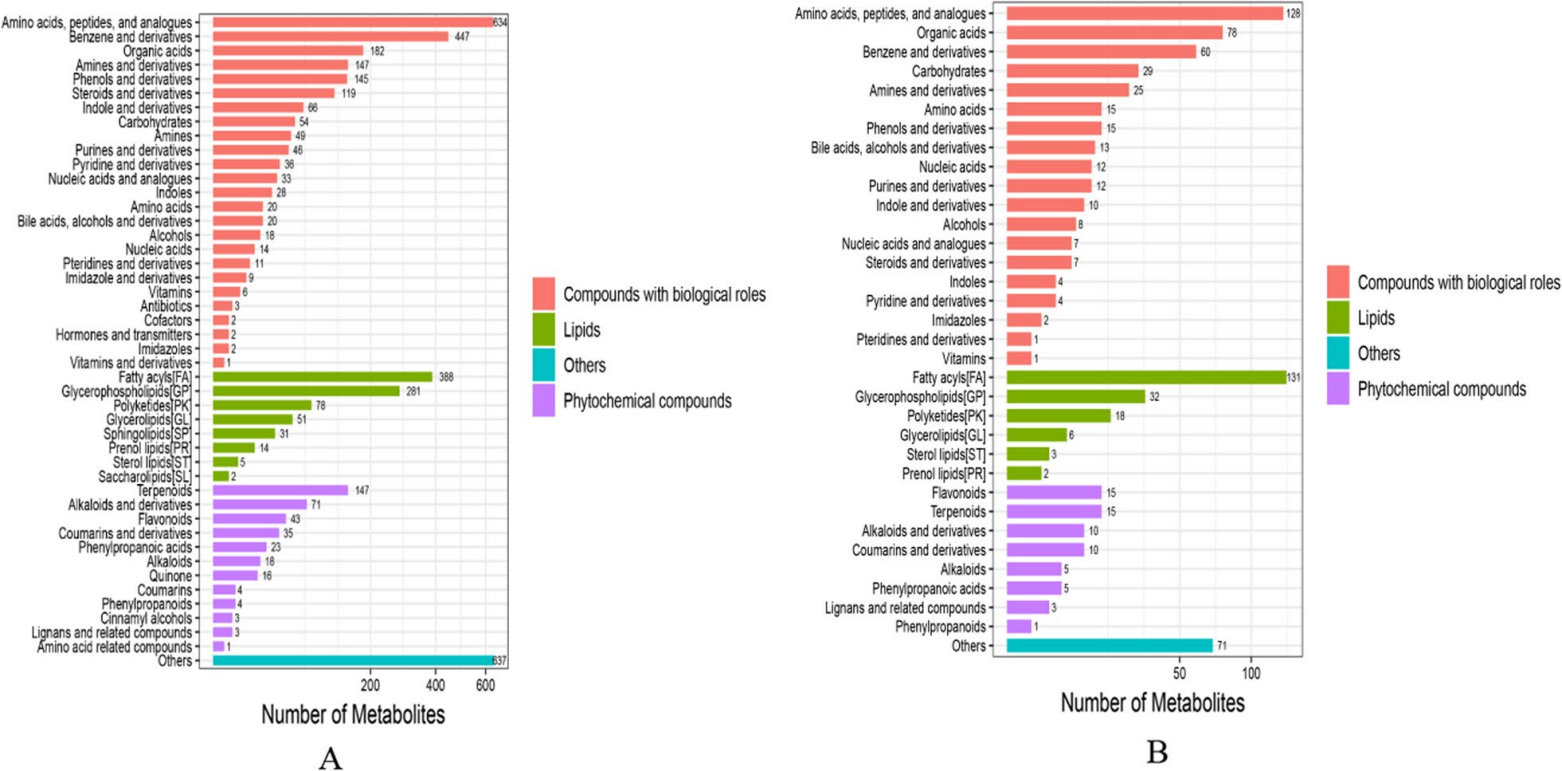
Classification annotations of differentially abundant metabolites in duck infection group compared with control group. **A** positive ion mode, **B** negative ion mode

The metabolites identified after comparing the TD group with the CK group were classified and annotated with reference to the KEGG and HMDB databases. Under the two ion modes, the identified compounds were divided into 4 categories, namely, biologically significant compounds, lipid compounds, phytochemicals and substances not yet classified in other categories. The biologically important compounds obtained in positive ion mode included amino acids, benzene, phenols, amines, steroids, indole, purine, imidazole, pyridine, and their derivatives, as well as organic acids, hormones, transfer factors, antibiotics, and vitamins. A total of 2094 biologically important metabolites 9included 850 lipids, 368 phytochemicals, and 637 unclassified metabolites (Fig. 4A). A total of 192 metabolites were lipid substances, 64 metabolites were plant compounds, and 71 were unclassified metabolites (Fig. 4B).

(2) Path annotation

**Fig. 5:**
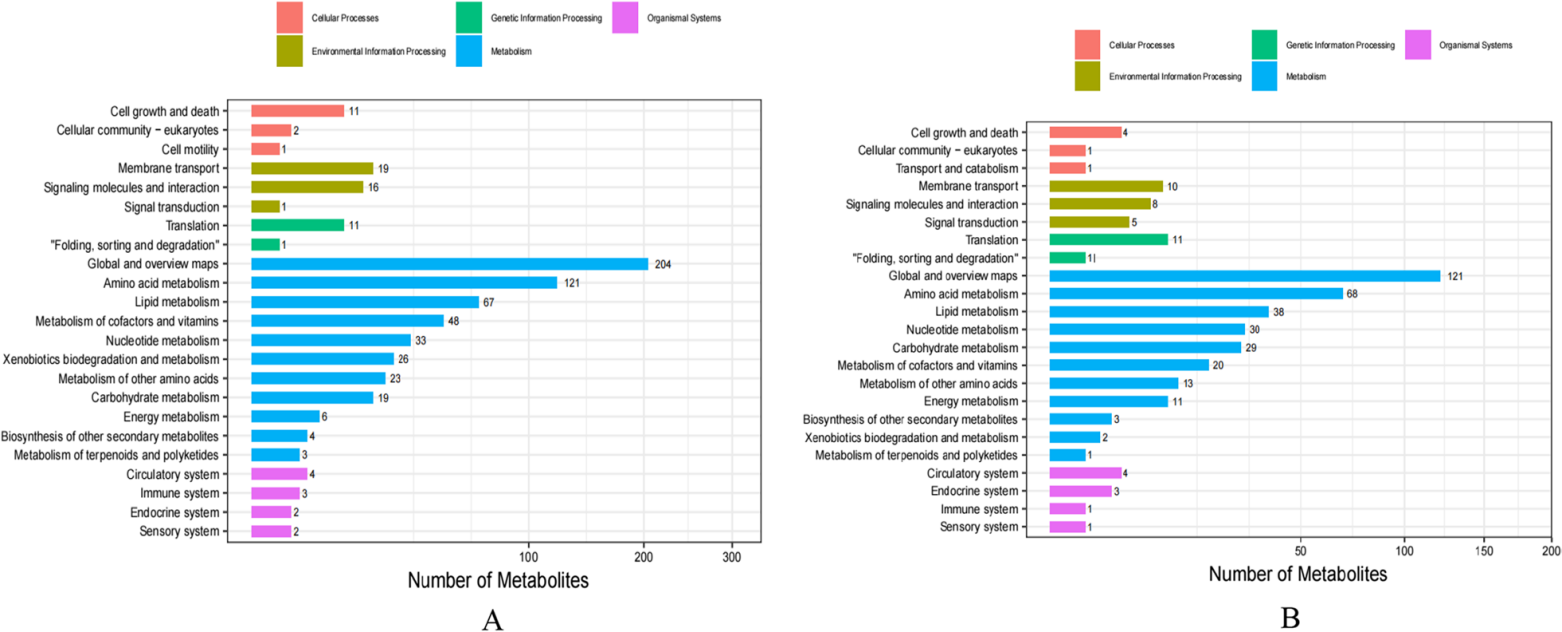
Differences in metabolite pathways between infected ducks and control ducks. **A** positive ion mode, **B** negative ion mode

The KEGG database was used for metabolite function annotations. To investigate the pathways involving various metabolites more deeply, the biochemical metabolic pathways and signal transduction pathways associated with the differentially abundant metabolites were analysed. Pathway diagrams and substance change trends were examined. Metabolic pathways are divided into 5 major functional branches: cellular processes, genetic information processing, organic systems, environmental information processing (environmental information processing), and metabolism (metabolism), and branches of differentially abundant ions in negative ion mode were consistent with those of the positive ions. The metabolic processes involving differentially abundant metabolites can be divided into 23 functional categories. In positive ion mode, differentially abundant metabolites participated in 627 pathways, with relatively few pathways involved in cellular process pathways and organic system systems, whereas global and global overview maps and the pathways of amino acid metabolism were the most abundant (Fig. 5A). The metabolic pathways in negative ion mode were consistent with those in positive ion mode, and the differentially abundant metabolites participated in 386 pathways (Fig. 5B).

#### Screening of differentially abundant metabolites

1. PCA

**Fig. 6:**
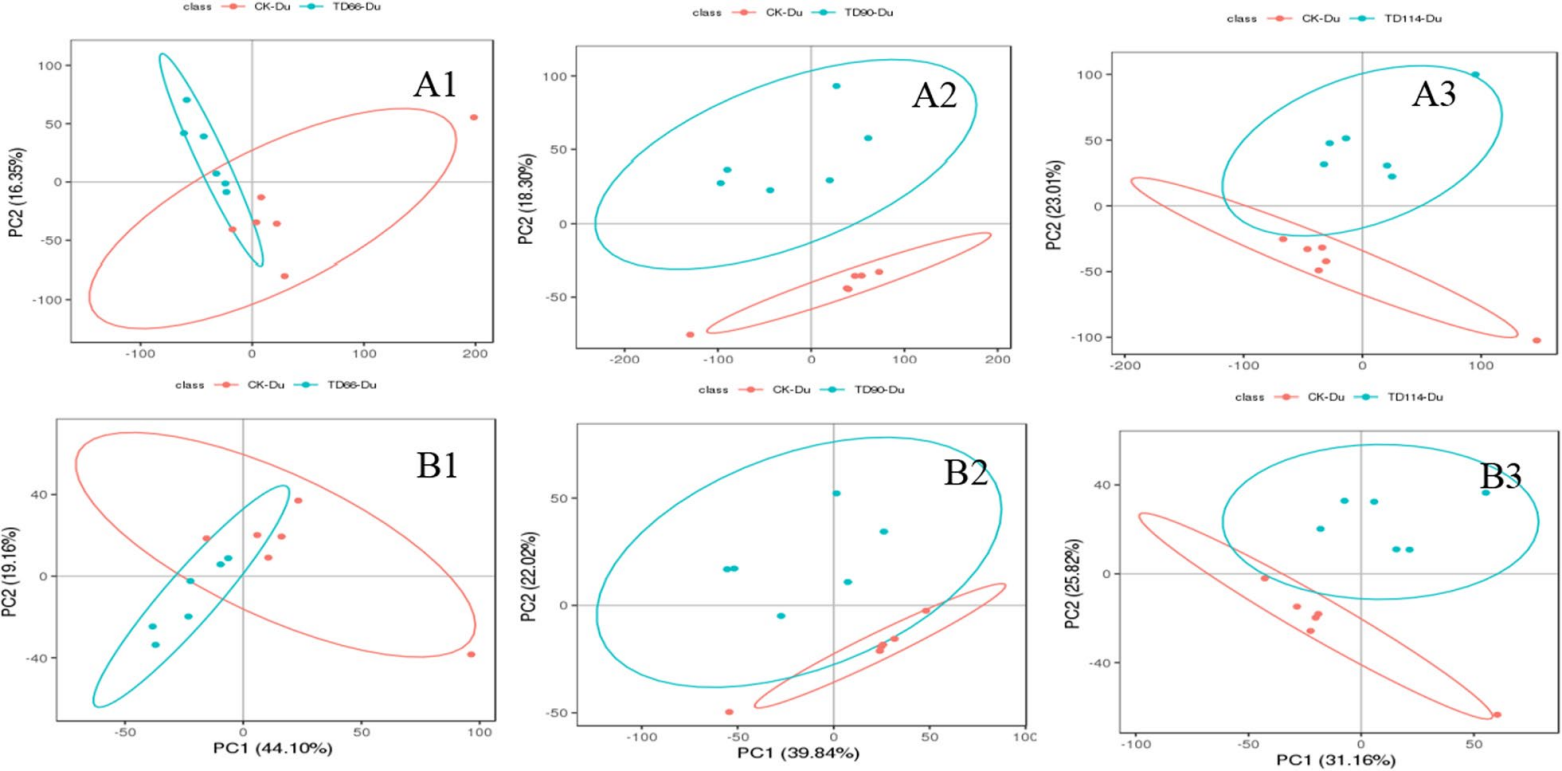
PCA results between the duodenumof the DEV infection and CK at different time periods. A1–A3 represent the comparison of DEV 66 h, 90 h, 114 h in positive ion mode with CK group; B1–B3 represent the comparison of DEV 66 h, 90 h, 114 h in negative ion mode with CK group

To distinguish the infection group from the control group, PCA was performed on the metabolite data to observe the distribution and separation trend of the two groups of samples. Before building the PCA model, log2 transformation was performed on the data, and the data were adjusted proportionally by Pareto scaling. For metabolomics research on the duodenum and ileum at different time points (66 h, 90 h, 114 h), the normal group was compared with the infected group to understand the degree of sample aggregation and dispersion between groups. The PCA results are as follows:

① The TD group duodenum samples were compared with those from the CK group. In PCA, the TD group formed a cluster, and there was no dispersion among the groups. One sample in the CK group deviated from the other 5 samples, and there was a partial overlap between the CK group and the TD group. The same ionic compounds existed in both (Fig. 6). In positive ion mode, there was no overlap between the CK group and the TD group at 90 h, and there were differences in compounds between the two groups, indicating that DEV infection significantly changed the metabolite profile at 90 h, and the subsequent additional changes might be attributable to the activation of the body's defence mechanism at 114 h, resulting in significant changes in differentially abundant metabolites from 90 h.

**Fig. 7:**
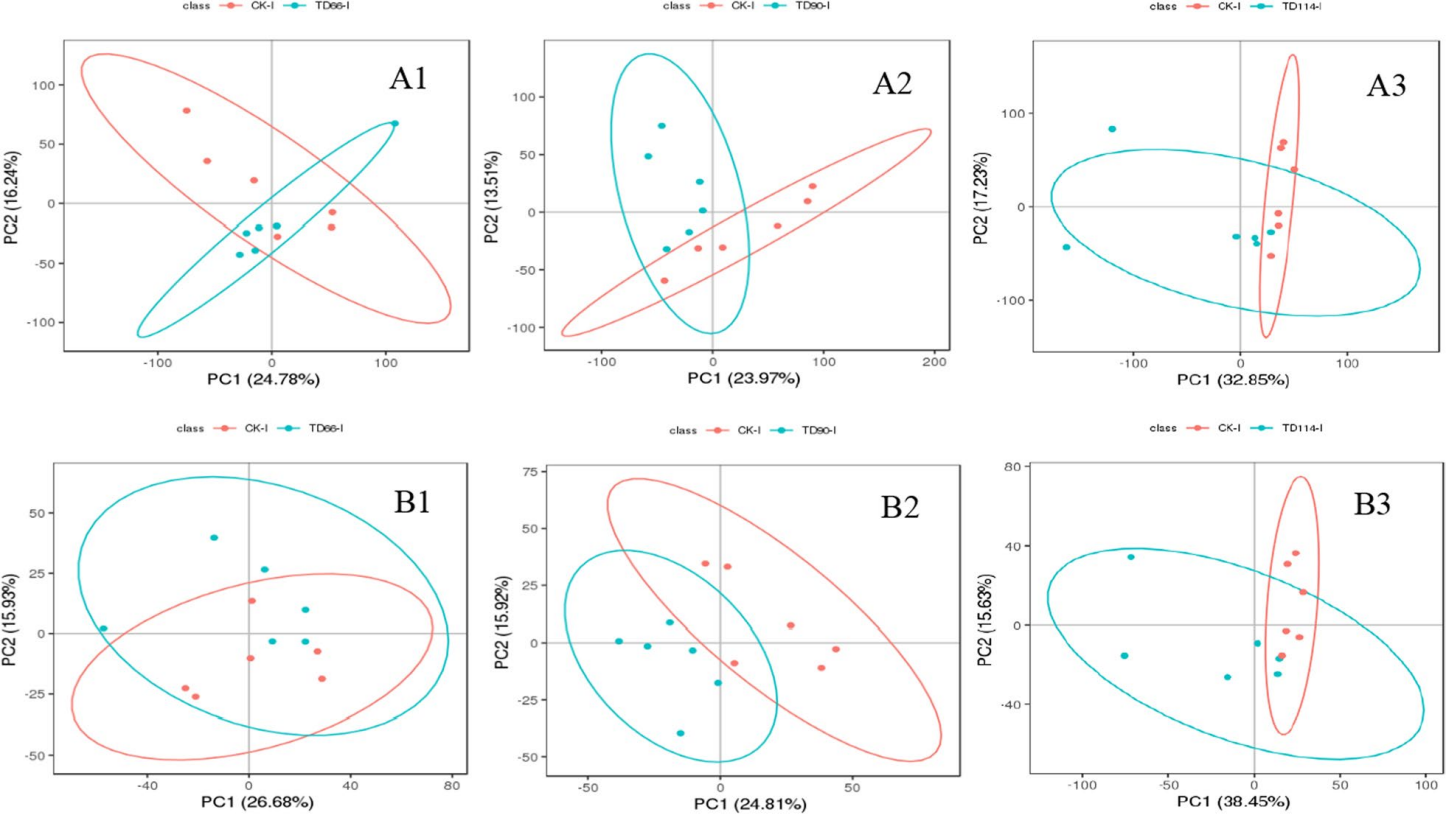
PCA results between the ileum of the DEV infection and CK at different time. A1–A3 represent the comparison of DEV 66 h, 90 h, 114 h in positive ion mode with the CK group; B1–B3 represent the comparison of DEV 66 h, 90 h, 114 h in negative ion mode with the CK group

② In the ileum of the CK group and the TD group, the PCA of the TD group formed a cluster, and there was no dispersion but some overlap between the groups, indicating that some compounds were the same between groups (Fig. 7). However, in positive ion mode, some samples deviated from the overall trend in the ileum of the infection group at 114 h, and two outliers were observed (Fig. 7A3).

The comprehensive PCA chart can distinguish the control group from the infection group; that is, DEV infection changes the metabolite profile of duck intestinal tissue, and there are differentially abundant compounds in the duodenum. Note that A1-A3 represent DEV 66 h, 90 h and 114 h in positive ion mode compared with the CK group; B1-B3 represent the comparison of DEV 66 h, 90 h, and 114 h in negative ion mode with the CK group.

(2) PLS-DA analysis

In the PLS-DA analysis, the Euclidean distance was used to determine the difference between groups. The dispersion between groups was obvious, indicating a greater difference [10]. To prevent the illusion of "overfitting" in the PLS-DA model under computer software, it is necessary to check the quality of the PLS-DA model using a 30-fold interactive verification, and the results are represented by parameters R2 and Q2; that is, to explain the model and prediction and verify the reliability of the PCA model, the cumulative explanation rate of the model is shown in Table 2. If the value of the model evaluation parameter (R2Y, Q2) is approximately l, or generally if Q2 is greater than 0.5, the model is stable and reliable. For 0.3 < Q2 < 0.5, the model stability is moderate, and for Q2 < 0.3, the model reliability is low. The information in Table 2 shows that the model interpretability is high, and the model is stable and reliable; that is, there are no overfitting "artefacts", and the established DEV infection model can be used for subsequent screening of differences.

**Table 2:** PLS-DA model parameters of different sample comparison groups under the two modes

Group	Ion mode	Model explanation rate	Model prediction rate
		R2Y (cum)	Q 2(cum)
TD66-Du-vs.-CK-Du	+	0.99	0.57
	–	0.97	0.74
TD66-I-vs.-CK-I	+	1	0.36
	–	1	0.38
TD90-Du-vs.-CK-Du	+	1	0.72
	–	0.99	0.76
TD90-I-vs.-CK-I	+	1	0.37
	–	1	0.54
TD114-Du-vs.-CK-Du	+	1	0.86
	–	0.98	0.82
TD 114—I -vs.-CK- I	+	0.98	0.45
	–	0.99	0.59

+: positive ion mode; –: negative ion mode

(3) Screening of the number of differentially abundant metabolites

Multivariate statistical analysis (PCA and PLS-DA) combined with univariate analysis (fold change, FC) and the t test (Student's t test) were used to screen differentially abundant metabolites between groups. PCA and PLS-DA were used to establish the relationship model between the expression of metabolites and the sample category to predictthe sample category in combination with the difference multiple and t test, and finally to identify the differentially abundant metabolites among the groups. The screening conditions for differentially abundant metabolites among groups were VIP ≥ 1 for the first two principal components of the PLS-DA model, *P* value < 0.05, FC > 1.2 or FC < 0.833. See Table 3 for the statistical results of the up- and downregulation of differentially abundant metabolites.

**Table 3:** Statistical table of differential compound detection

Group	Ion mode	Increased quantity	Reduced quantity	Different quantity
Group	Mode	Up	Down	Different
TD66-Du-vs.-CK-Du	+	1127	442	1569
	−	274	129	403
TD90-Du-vs.-CK-Du	+	596	1060	1656
	−	159	246	405
TD114-Du-vs.-CK-Du	+	867	1009	1876
	−	216	228	444
TD66-I-vs.-CK-I	+	340	359	699
	−	117	74	191
TD90-I-vs.-CK-I	+	491	573	1064
	−	187	136	323
TD 114—I -vs.-CK- I	+	464	725	1189
	−	197	184	381

+: positive ion mode; −: negative ion mode

It can be seen from Table 3 that compared with the control group, the total number of differentially abundant compounds identified by screening of the duodenum at 66 h, 90 h, and 114 h after DEV infection gradually increased, especially in positive ion mode, with 1569, 1656 and 1876 differentially abundant compounds. In negative ion mode, the TD group had fewer differences than the CK group, with 403, 405 and 444 differentially abundant compounds. While the differences in compounds in the ileum were not as large as those in the duodenum, the overall metabolism changed significantly.

(4) Duodenal differentially abundant compound screening

The differentially abundant compounds in Table 3 were defined as follows: FC is greater than or equal to 1.2 or less than or equal to 0.833; the *P* value indicates significance (P < 0.05); and VIP is greater than or equal to 1. The metabolites detected by the LC‒MS analysis platform are all consistent with the standard. The metabolites in the product database are matched at the first and second levels, and the final difference is obtained by referring to the existing numbers in the KEGG Database or the HMDB.

Compared with the CK-Du group, the TD66-Du group contained 41 significantly differentially abundant compounds; in positive ion mode, there were 27, and in negative ion mode, there were 14. Among these, 18 were increased in abundance, and 23 kinds were decreased. Compared with the CK-Du group, the TD90-Du group contained 52 significantly differentially abundant compounds; among them, there were 33 differentially abundant substances in positive ion mode and 19 differentially abundant substances in negative ion mode, and 10 were increased in abundance, while 42 differentially abundant substances were decreased. Compared with the CK-Du group, the TD114-Du group contained 64 significantly differentially abundant compounds: 38 in positive ion mode, 26 in negative ion mode, 22 increased in abundance, and 22 decreased in abundance. The infection group had 42 differentially abundant metabolites in common at all three time points (66 h, 90 h, 114 h). To further screen for important differentially abundant metabolites, in the range of *P* value < 0.01 & VIP ≥ 2, the metabolites were sorted by logFC value (for logFC greater than 1, larger values are more meaningful; for logFC less than − 1, smaller values are more meaningful). According to the above screening results, the number of significantly differentially abundant metabolites in the duodenum gradually increased from 2 to 12 to 22 as the time increased from 66 to 90 h and then to 114 h. The metabolites cytidine, 2'-deoxynucleoside and 4-guanidinobutyric acid were common differentially abundant metabolites at all three points.

(5) Screening of differentially abundant compounds in the ileum

Compared with the CK-I group, the TD66-I group contained 16 significantly differentially abundant compounds. There were 10 differentially abundant substances in positive ion mode and 6 differentially abundant substances in negative ion mode. Among them, 14 were increased in abundance and 14 were decreased in abundance. Compared with the control group, the TD90-I group contained 52 identified differentially abundant compounds compared with the CK-I group, 32 substances in positive ion mode, and 20 in negative ion mode, of which 24 were increased in abundance and 28 were decreased in abundance. The TD114-I group contained 40 significantly differentially abundant compounds compared with the CK-I group, 21 in positive ion mode and 19 in negative ion mode, of which 11 substances had a contribution value greater than 1, and 29 substances had a contribution value less than 1. The infection group had the same differentially abundant metabolites at different time periods (66 h, 90 h, 114 h) compared with the CK group. To further screen for meaningful differentially abundant metabolites, within the range of *P* value < 0.01 & VIP > 2, the metabolites were sorted by logFC value (for logFC greater than 1, larger values are more meaningful; for logFC less than − 1, smaller values are more meaningful). According to further screening, the number of significant differentially abundant metabolites in the ileum gradually increased 2 at 66 h to 8 at 90 h and then to 11 at 114 h, and the number of types of differentially abundant metabolites was smaller than that in the duodenum.

#### Heatmap analysis of differentially abundant metabolites

To visualize the differences and perform cluster analysis between the control group and the infected group, the differentially abundant substances obtained above were imported into the pheatmap in the pheatmap package for scaling to obtain a heatmap. At 66 h, 90 h and 114 h, cluster analysis of the differentially abundant metabolites in the duodenum and ileum of the DEV-infected group and the control group was performed and presented in combination with a heatmap.

**Fig. 8:**
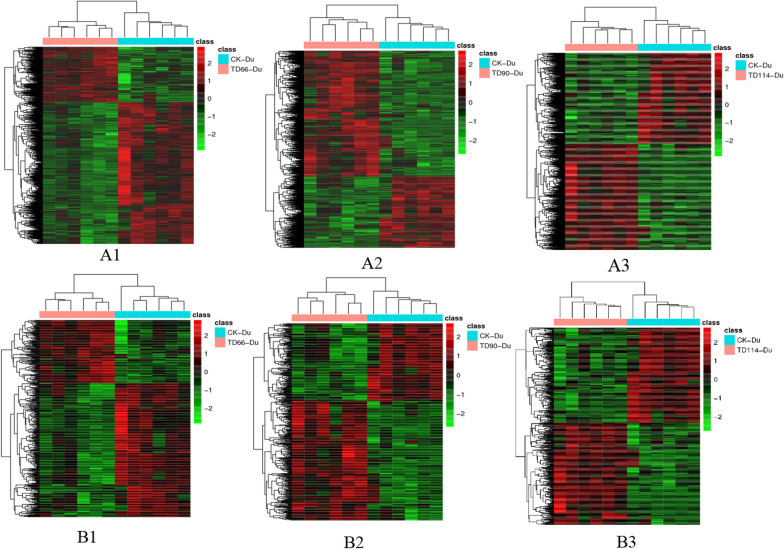
Heatmap of differentially abundant metabolites between experimental and control duodenums at different time periods. **A1**, **A2**, **A3**: comparison between TD66, TD90, TD114 and CK in positive ion mode; **B1**, **B2**, **B3**: comparison between TD66, TD90, TD114 and CK in negative ion mode. Each row in the figure represents a differentially abundant ion. Each column represents a sample, different colours represent differences in abundance, and the colour range from green to red, representing low to high differences

① There was an overall difference in the expression of metabolites between the TD-Du and CK-Du groups when viewed horizontally (the colour difference between the two groups was more obvious), and the number of rows was greater, indicating a larger number of differentially abundant metabolites. In positive ion mode, the differential abundance in TD-Du compared to CK-Du changed from higher at 66 h to lower at 90 h, as reflected by the change from red to green. At 114 h, an increased number of differentially abundant substances were found. The differences in samples between infection groups as well as between the infection and CK group caused the colour classification to be blurred. Relatively speaking, the differences within each group were small (Fig. 8A1, A2, A3). In negative ion mode, the situation was generally consistent with that in positive ion mode, but longitudinal analysis showed that TD90-Du has decreasing numbers of differentially abundant metabolites compared to CK-Du over time (Fig. 8B1, B2, B3).

**Fig. 9:**
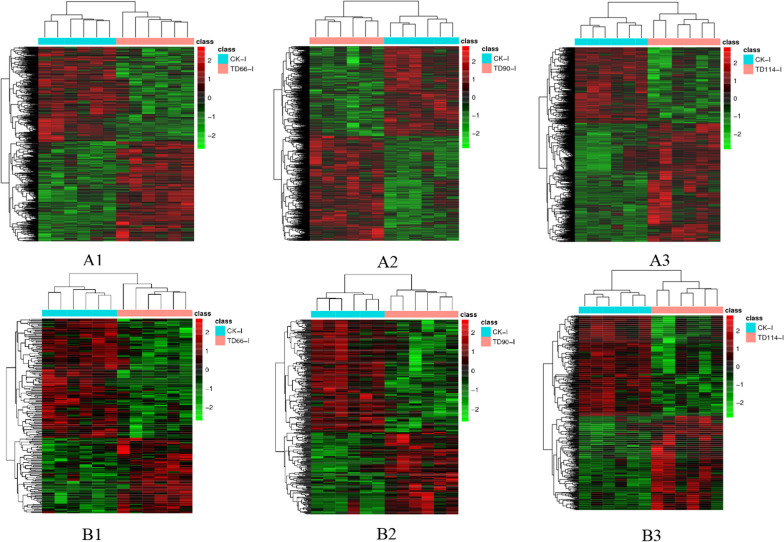
Heatmap of differentially abundant metabolites between experimental and control (CK) ileum at different time points. **A1**, **A2**, **A3**: comparison between TD66, TD90, TD114 and CK in positive ion mode; **B1**, **B2**, **B3**: comparison between TD66, TD90, TD114 and CK in negative ion mode. Each row in the figure represents a differentially abundant ion. Each column represents a sample. Different colours represent differences in abundance, and the colour range from green to red represent low to high differences

② Compared with CK-I, TD-I showed differences in the abundance of metabolites in the horizontal direction (the colour difference between the two groups was more obvious), and the number of rows was greater, indicating a larger number of differentially abundant metabolites. In positive ion mode, the abundance of a number of metabolites compared to that in CK-I changed from higher at 66 h to lower at 114 h, reflected in a colour change from red to green. The comparison of the TD-I and CK-I groups at 90 h revealed an increased number of differentially abundant substances. CK-I and TD-I showed improved clustering. The overall colour change within each group was not large, but there were differences in samples between the infection groups and the CK group. The colour classification is relatively fuzzy when the difference within the group is small (Fig. 9A1, A2, A3). In negative ion mode, the overall situation was generally consistent with that in positive ion mode, and the number of differentially abundant metabolites in TD-I compared to CK-I increased with later time points (Fig. 9B1, B2, B3).

#### Metabolic pathway analysis of differentially abundant metabolites

1. Metabolic pathway analysis of differentially abundant metabolites in the duodenum

In positive ion mode, differentially abundant metabolite pathway analysis of the TD66- Du group, TD90- Du group, TD114- Du group and CK- Du group showed that the pathways in which the differentially abundant metabolites participated were consistent and that the number of pathways was consistent with the number of differentially abundant metabolites. The pathways were sorted according to the number of differentially abundant metabolites involved (number ≥ 10) as follows: metabolism, amino acid biosynthesis, tryptophan metabolism, tyrosine metabolism, purine metabolism, ABC transporters, cysteine and methionine metabolism, phenylalanine metabolism, neuronally active ligand-receptor interactions, cytochrome P450 metabolism xenobiotics, arginine and proline metabolism, 2-oxocarboxylic acid metabolism, steroid hormone synthesis, nicotinate and nicotinamide metabolism, aminoacyl tRNA biosynthesis, pyrimidine metabolism, glycine, serine and threonine metabolism, lysine degradation, histidine metabolism, ferroptosis, carbon metabolism, etc.

In negative ion mode, differentially abundant metabolite pathway analysis of the TD66-Du group, TD90-Du group, TD114-Du group and CK- Du group showed that the pathways involving the differentially abundant metabolites were consistent, and the pathways were sorted according to the number of differentially abundant metabolites involved (Number ≥ 10) as follows: metabolism, amino acid biosynthesis, purine metabolism, carbon metabolism, 2-oxocarboxylic acid metabolism, unsaturated fatty acid biosynthesis, aminoacyl tRNA biosynthesis, arginine and proline metabolism, phenylalanine metabolism, ABC transporters, glyoxylate and dicarboxylate metabolism, cysteine and methionine metabolism, alanine, aspartate and glutamate metabolism, pyrimidine metabolism, tyrosine metabolism, etc.

**Fig. 10:**
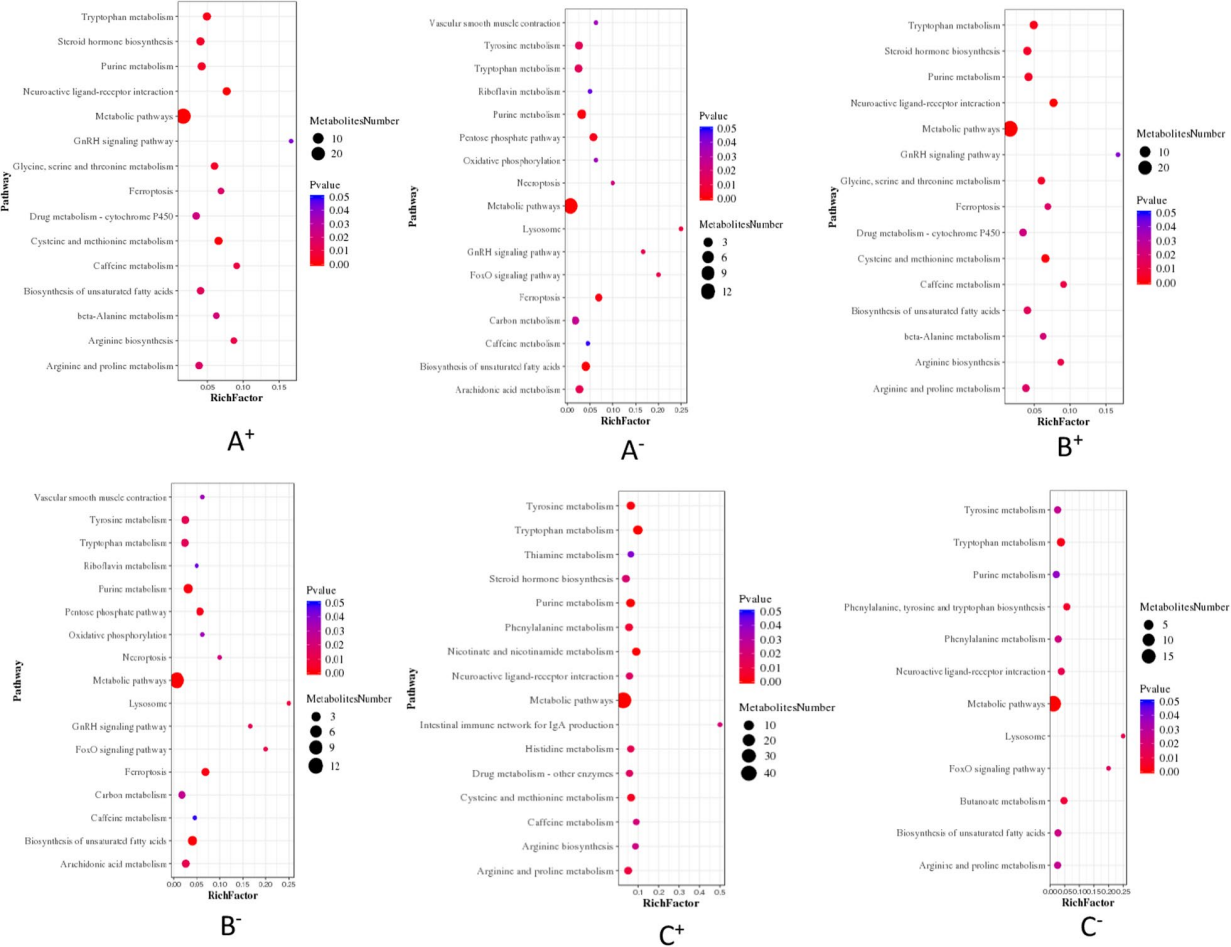
Enrichment bubble chart of metabolic pathways with significant differences between TD-Du and CK-Du. A, TD66-Du group; B, TD90-Du group; C, TD114-Du; Note: + positive ion mode, −negative ion mode

In positive ion mode, pathway enrichment analysis of the differentially abundant metabolites in the TD66-Du group and the CK-Du group, was performed with a significance threshold of *P* < 0.05, and pathways were ranked by the number of differentially abundant metabolites annotated to the pathway. The top four were metabolism, neuroactive ligand‒receptor interaction, cysteine and methionine metabolism, and tryptophan metabolism. The largest enrichment factor of differentially abundant metabolites was in the lysosomal pathway (Fig. 10A^+^). In negative ion mode, the top four pathways were metabolism, biosynthesis of unsaturated fatty acids, purine metabolism and ferroptosis, and the largest enrichment factor of differentially abundant metabolites was likewise in the lysosome pathway (Fig. 10A^−^).

In positive ion mode, enrichment analysis of the pathways involved in the differentially abundant metabolites of the TD90-Du group and the CK-Du group, was performed, and sorting was performed according to the number of differentially abundant metabolites (P < 0.05) annotated to the pathways. The top four were metabolism, amino acid biosynthesis, ABC transporter and purine metabolism, and the largest enrichment factor of differentially abundant metabolites was in the intestinal immune network pathway formed for the production of IgA (Fig. 10B^++^). In negative ion mode, the top four pathways were metabolism, 2-oxocarbonyl acid metabolism, amino acid biosynthesis and carbon metabolism, and the largest enrichment factor of differentially abundant metabolites was in the lysosome pathway (Fig. 10B^−^).

In positive ion mode, enrichment analysis of the pathways involving the differentially abundant metabolites between the TD114-Du group and the CK-Du group was performed, and the pathways were sorted according to the number of differentially abundant metabolites (P < 0.05) annotated to each pathway. The top four were metabolism, tryptophan metabolism, purine metabolism and tyrosine metabolism, and the largest enrichment factor of differentially abundant metabolites was in the intestinal immune network pathway formed for the production of IgA (Fig. 10C^+^). In negative ion mode, the top four pathways were metabolism, purine metabolism, tryptophan metabolism, and arginine and proline metabolism, and the largest enrichment factor of differentially abundant metabolites was in the lysosomal pathway (Fig. 10C^−^).

(2) Analysis of metabolic pathways of differentially abundant metabolites in the ileum

In positive ion mode, pathway analysis of the differentially abundant metabolites of the TD66-I group, TD90-I group, TD114-I group and CK-Du group showed that the pathways involving the differentially abundant metabolites were consistent. The pathways were sorted according to the number of differentially abundant metabolites involved (number ≥ 10) as follows: metabolic pathways, amino acid biosynthesis, tryptophan metabolism, tyrosine metabolism, purine metabolism, ABC protein transport, cysteine and methionine metabolism, phenylalanine metabolism, Neuroactive ligand‒receptor interactions, cytochrome P450 metabolism xenobiotics, arginine and proline metabolism, 2-oxocarboxylic acid metabolism, steroid hormone synthesis, nicotinate and nicotinamide metabolism, aminoacyl tRNA biosynthesis, pyrimidine metabolism, glycine, serine and threonine metabolism, lysine degradation, histidine metabolism, ferroptosis, carbon metabolism.

In negative ion mode, differentially abundant metabolite pathway analysis of the TD66-I group, TD 90-I group, TD114-I group and CK-Du group showed that the pathways in which the differentially abundant metabolites participated were consistent, and the pathways were sorted according to the number of differentially abundant metabolites involved (number ≥ 10) as follows: metabolic pathways, biosynthesis of amino acids, purine metabolism, carbon metabolism, 2-oxocarboxylic acid metabolism, biosynthesis of unsaturated fatty acids, biosynthesis of aminoacyl tRNA, arginine and Proline metabolism, phenylalanine metabolism, ABC protein transport, glyoxylate and dicarboxylate metabolism, cysteine and methionine metabolism, alanine, aspartate and glutamate metabolism, pyrimidine metabolism, tyrosine metabolism.

**Fig. 11:**
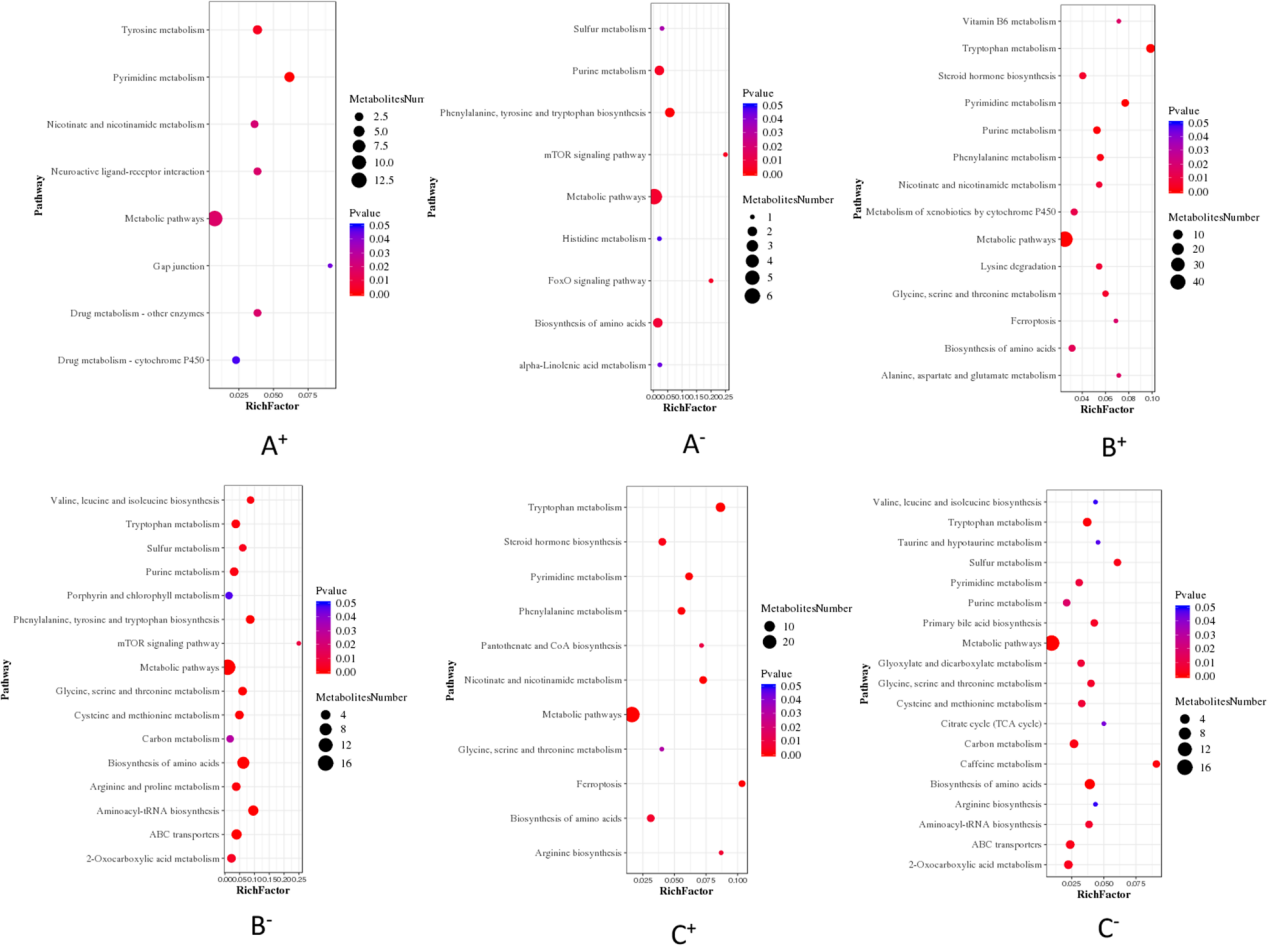
Enrichment bubble diagram of metabolic pathways with significant differences between TD-I and CK-I. **A** TD66-I group; **B** TD90-I group; **C** TD114-I; Note: + positive ion mode, −negative ion mode

In positive ion mode, enrichment analysis of the pathways involved in the differentially abundant metabolites of the TD66-I group and the CK-I group was performed, and the results were sorted by the number of differentially abundant metabolites (P < 0.05) annotated to the pathways. The top four were metabolic pathways, pyrimidine metabolism, tyrosine metabolism and drug metabolism-cytochrome P450, and the largest enrichment factor of differentially abundant metabolites was in the gap junction pathway (Fig. 11A^+^). In negative ion mode, the top four metabolic pathways were phenylalanine, tyrosine and tryptophan biosynthesis, purine metabolism and amino acid biosynthesis, and the mTOR signalling pathway had the largest differentially abundant metabolite enrichment factor (Fig. 11A^−^).

In positive ion mode, enrichment analysis of the pathways involved in the differentially abundant metabolites of the TD90-I group and the CK-I group was performed, and the results were sorted according to the number of differentially abundant metabolites (*P* < 0.05) annotated to the pathways. The top four were metabolic pathways, tyrosine metabolism, pyrimidine metabolism and purine metabolism, and the largest enrichment factor of differentially abundant metabolites was in the tryptophan metabolism pathway (Fig. 11B ^+^). In negative ion mode, the top four were metabolic pathways, amino acid biosynthesis, ABC transporter and aminoacyl-tRNA biosynthesis, and the mTOR signalling pathway had the largest enrichment factor of differentially abundant metabolites (Fig. 11B^−^).

In positive ion mode, enrichment analysis of the pathways involved in the differentially abundant metabolites of the TD114-I group and the CK-I group was performed, and the results were sorted according to the number of differentially abundant metabolites (*P* < 0.05) annotated to the pathways. The top four were metabolic pathways, tryptophan metabolism, biosynthesis of amino acids, and steroid synthesis, and the largest enrichment factor for differentially abundant metabolites was in the ferroptosis pathway (Fig. 11C^+^). In negative ion mode, the top four were metabolic pathways, 2-oxocarbonyl acid metabolism, amino acid biosynthesis, and ABC transporters, and the caffeine metabolic pathway had the largest enrichment factor for differentially abundant metabolites (Fig. 11C^−^).

## Discussion

Duck enteritis virus (DEV) infection mainly causes ducks, geese and other Anseriformes to develop characteristic clinical symptoms, such as body cavity haemorrhage, intestinal inflammation and parenchymal organ degeneration [11]. At present, to explore its pathogenesis, it is necessary to establish an infection model. Generally, the establishment of the model involves simulating natural infection, and the quality of the model must then be judged. In this study, a common PCR identification method and a PLS-DA model prediction method were used for this purpose, and the high predictability values obtained from each PLS-DA model indicated effective simulation of natural infection. Metabolomics is an emerging field of systems biology in which analytical chemistry methods are used to explore endogenous small-molecule metabolites in tissues or biological fluids. In terms of metabolomics research techniques, LC‒MS has become the next step of GC‒MS and the main technological platform after 1H NMR metabolic profiling [12, 13]. Metabolomics has been applied to the study of various viral infections, examining the characteristics of human patient body fluid or tissue samples, clinical animal models of viral infection, and electronic models of viral metabolic networks [13, 14]. In this study, untargeted metabolomics technology was used to examine the changes in intestinal metabolites and related metabolic pathways in DEV-infected ducks at 66 h, 90 h, and 114 h compared with the control group at 66 h. With increasing infection time, increasing numbers of differentially abundant metabolites were observed, and related metabolic pathways are increasingly enriched.

In this study, the intestinal metabolites of DEV-infected ducks were analysed using multivariate statistical analyses, such as PCA and PLS-DA, and the results showed that the metabolite profiles differed significantly between the CK group and the DEV group. Classification of the differentially abundant metabolites showed that they were predominantly in physiological activities such as amino acid metabolism, exogenous biodegradation and lipid metabolism, cell growth and death, signal transduction, and the regulation of the immune system. These results are consistent with the findings of Zhang et al. [15], who determined that DEV infection changes signalling pathways, the production of IgA in the intestinal immune network, and smooth muscle activity and regulates a-linolenic acid metabolism, glycine, serine and threonine metabolism and other metabolic pathways and signalling pathways. Consistently, amino acid metabolism plays a nonnegligible role in this process. Amino acids are important for organisms because they can not only be used in the synthesis of proteins and other biologically active molecules but also provide raw materials for the synthesis of most cytokines. Many recent metabolomic studies have found that viral infection can increase the synthesis of amino acids in organisms 16. Similar results were obtained in this experiment: DEV infection increased the production of lysine, tryptophan, citrulline, histidine, methionine, serine, ornithine, and threonine.

Tryptophan (Trp) catabolism is considered to be an important factor in inflammation and the immune response [17], and various Trp metabolites, such as kynurenic acid (Kyn) [18], 5-serotonin (5-hydroxytryptamine) [19] and indole-3-carboxylic acid (IALD) [20], contribute to beneficial functions. Tryptophan is considered to have a positive role in reducing intestinal permeability and the expression of proinflammatory cytokines in IBS and is also considered to be a promising therapeutic candidate for the treatment of IBS [21]. In the present study, increased levels of tryptophan indicated a shift in metabolism towards normal levels, possibly due to further remission of IBD, which may be partly related to the addition of phytochemicals.

Methionine (Met) and cysteine (Cys) are sulfur-containing amino acids involved in various physiological functions. For example, they regulate intestinal functions such as digestion and metabolism of nutrients and mucosal resistance by maintaining the integration of the epithelial layer [22]. Therefore, after DEV infected the intestinal tract, methionine and cysteine were present, but there were no differential changes in methionine, and cysteine was found only at 114 h also in the form of N-acetyl-l-cysteine. N-acetylcysteine (NAC) is a powerful antioxidant and free radical scavenger, so it has a protective effect on the peroxidation of cell membrane lipids [23]. At 114 h, the level of N-acetyl-l-cysteine, which is the precursor of the tripeptide glutathione (GSH in the methionine cycle, was higher than that in the control group. Glutathione is the main cellular antioxidant in mammals and is closely related to free radical formation and lipid peroxidation [24]. Sulfur-containing amino acids, especially cysteine, play a key role in the cellular redox function of the digestive tract, and the protective role of cysteine is well known because it is the rate-limiting amino acid for glutathione synthesis [24, 25]. The application of methionine significantly increased the number of cells in the lamina propria, which may indicate a proinflammatory effect, and cysteine and N-acetyl-L-cysteine also exert anti-inflammatory effects [26]. L-cysteine and N-acetyl-L-cysteine have potential as effective drugs for the treatment of IBD to reduce inflammation and tissue damage [27]. At 114 h, N-acetyl-l-cysteine in the duodenum of DEV-infected ducks was elevated, possibly due to the influence of inflammatory factors on the intestinal wall cells of the duck at this time. The duck may have been compensating for the effects of DEV invasion by producing N-acetyl-l-cysteine to protect the intestinal mucosa.

Linoleic acid (LA), which can be found in the duodenum after DEV infection at 114 h, is an unsaturated fatty acid with an inhibitory effect on the inflammatory response, and its metabolites are related to cancer and various biological functions [28]. Fatty acids (FAs) are necessary for the normal functioning of all organisms; their structure can be changed by extension and desaturation, and their biological effects depend on the number of unsaturated bonds in their molecules [29]. Recent studies have found that unsaturated fatty acids have significant anti-inflammatory effects, confirming that NF-κB and NLRP 3 inflammasomes are involved in the pathological process of inflammation [30]. Arachidonic acid (AA, a fatty acid) can produce a variety of active metabolites (such as prostaglandin E, thromboxane, leukotrienes) [31]. These active metabolites can enhance vascular permeability and cause tissue oedema, increase the activity of adhesion molecules, promote the migration of inflammatory cells out of the blood vessel wall, and enhance the chemotactic response of monocytes and neutrophils, suggesting that AA plays an important role in the inflammatory response [32]. Prostaglandin G2 showed a downward trend in both groups, which may be due to the easy conversion of a large amount of prostaglandin G2 into other more effective inflammatory factors (such as prostaglandin H2 and prostaglandin E2). Another possible reason is that there is competition between prostaglandin G2 and AA. Prostaglandins can be found in the duodenum and ileum, but in different forms. In the duodenum, prostaglandins take the form of PGA2; in the ileum, prostaglandins take the form of PGB1. Although the names are similar, the roles of PGA2 are different: PGA2 induces apoptosis [33], and role of PGB1 has not yet been reported.

In the results of these experiments, there were fewer differentially abundant metabolites in the ileal segment and more differentially abundant metabolites in the duodenal segment, which may be related to the fact that substances are metabolized in the body by passing first through the duodenum, which is performs the function of digesting and absorbing nutrients, so that few substances reach the ileum.

## Conclusion

In this experiment, we successfully established a duck DEV infection model, which has good predictability and supervision and can meet the needs of subsequent metabolomics data determination. After comparative analysis, the number of differentially abundant metabolites in the duodenum and ileum after DEV infection differed to some extent, with the duodenal tissues having significantly more differentially abundant metabolites and the ileal tissues having fewer differentially abundant metabolites. The number of differentially abundant metabolites changed over time after DEV infection. The highest number of differentially abundant metabolites was found at 114 h of infection, followed by 90 h of infection, and the lowest was found at 66 h of infection. The same differentially abundant metabolites were present at all time points: prostaglandins, arachidonic acid and ethanolamine arachidonic acid, among others. The main metabolic pathways in the duodenum were the IgA-associated intestinal immune network pathway and the lysosomal pathway, and the metabolic pathways that were more enriched in the ileum included the mTOR signalling pathway, the ferroptosis pathway, and the tryptophan metabolism pathway.

### Supplementary Information

**Additional file 1.** BPC chart.
