# Supplementary material for: Untargeted metabolomics of the intestinal tract of DEV-infected ducks
Source: Virol J. 2023 Dec 19;20:305. doi: 10.1186/s12985-023-02266-x (PMC10731684; doi:10.1186/s12985-023-02266-x)

**Picture of the original PCR gel electrophoresis**


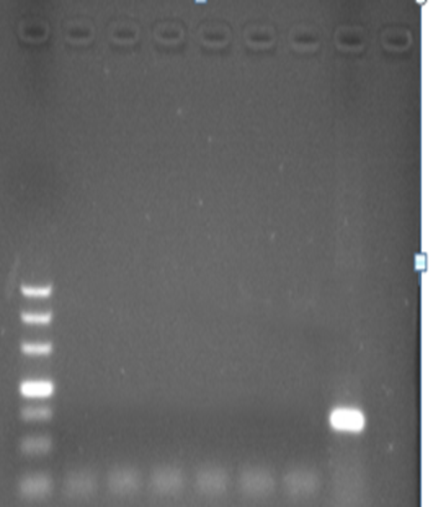


M 1 2 3 4 5 6 + -

bp

A

500

400

300

200

150

100

50

133bp


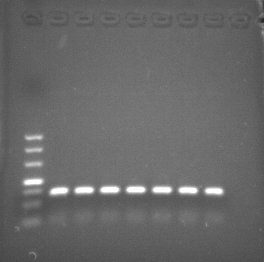


M 1 2 3 4 5 6 + -

bp

B

500

300

150

50

400

100

133bp

200

400

150


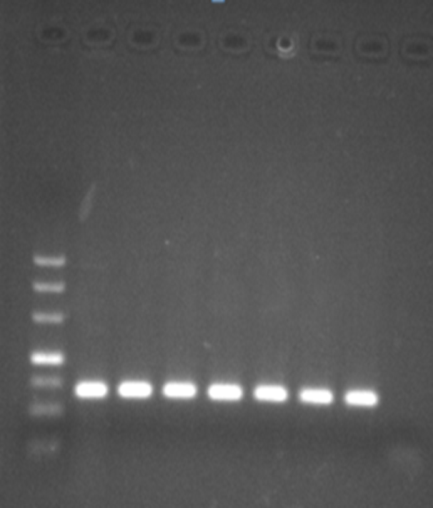


M 1 2 3 4 5 6 + -

bp

C

133bp

500

300

200

100

50


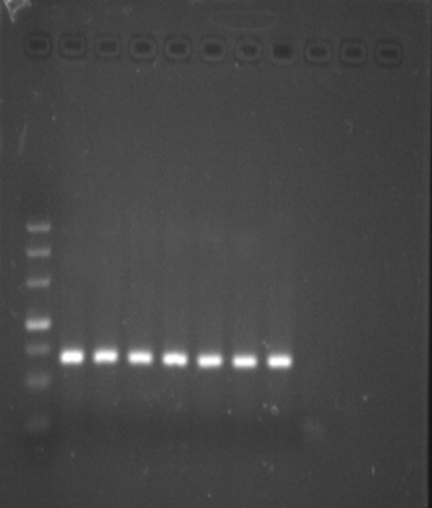


M 1 2 3 4 5 6 + -

500

bp

D

133bp

400

200

100

50

300

150

Fig. 2 Detection results of DEV-NP PCR in mixed duck tissues

A: Control group; B: DEV 66 h; C: DEV 90 h; D: DEV 114 h

Supplementary file of BPC peak maps for the remaining groups (images in ZIP)

CK-Du_BPC_neg


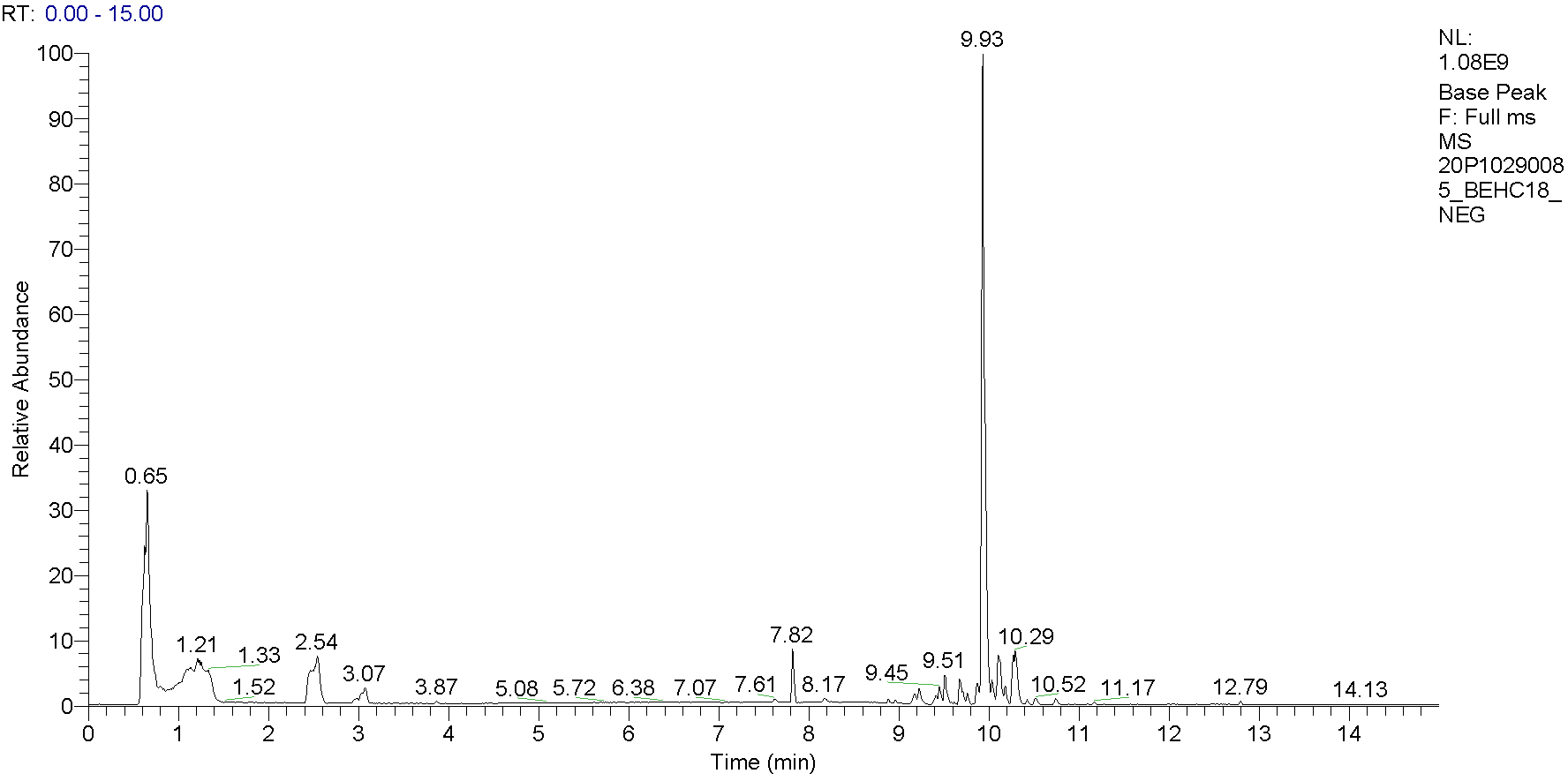


CK-Du_BPC_pos


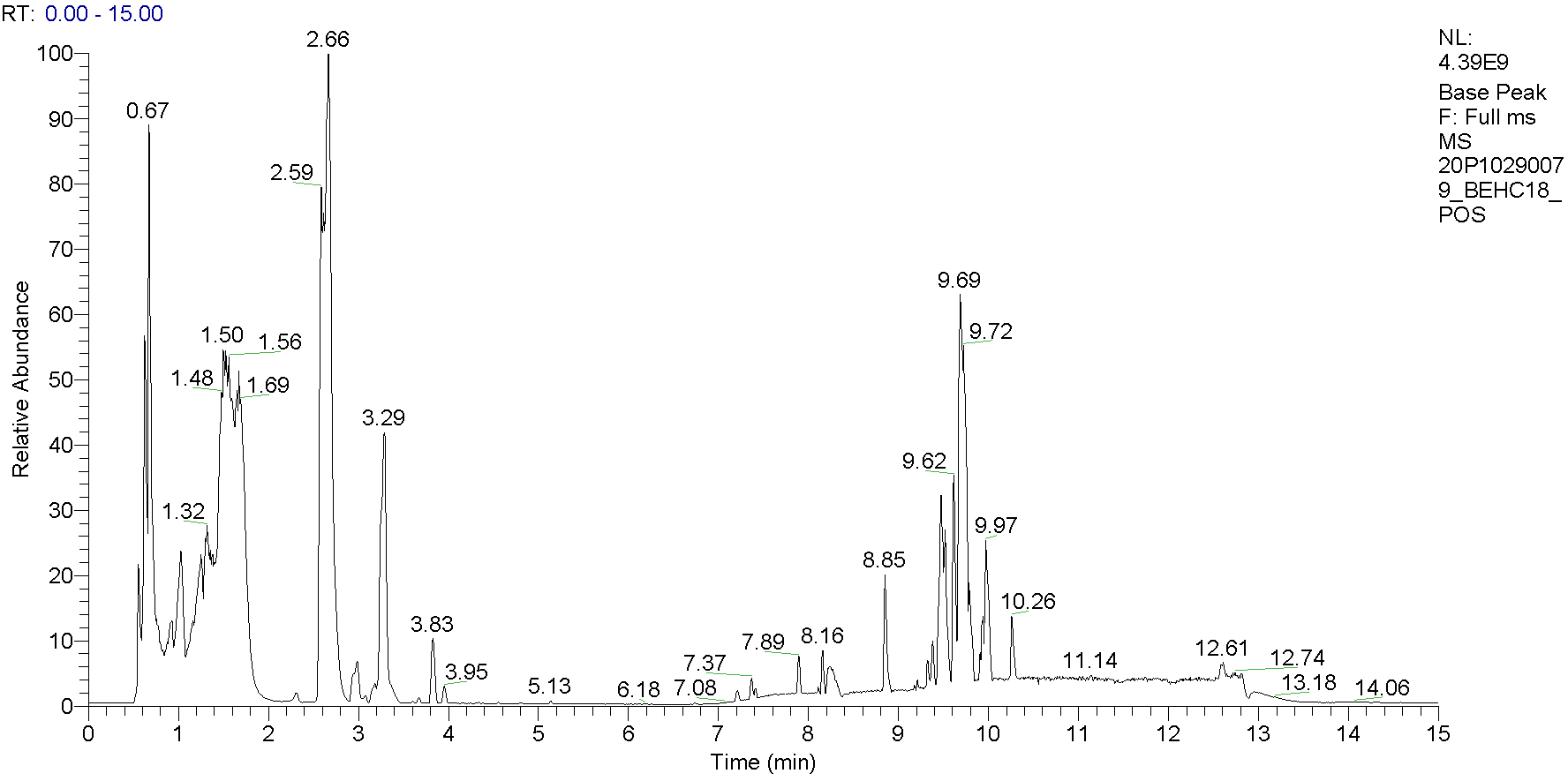


TD66-Du_BPC_neg


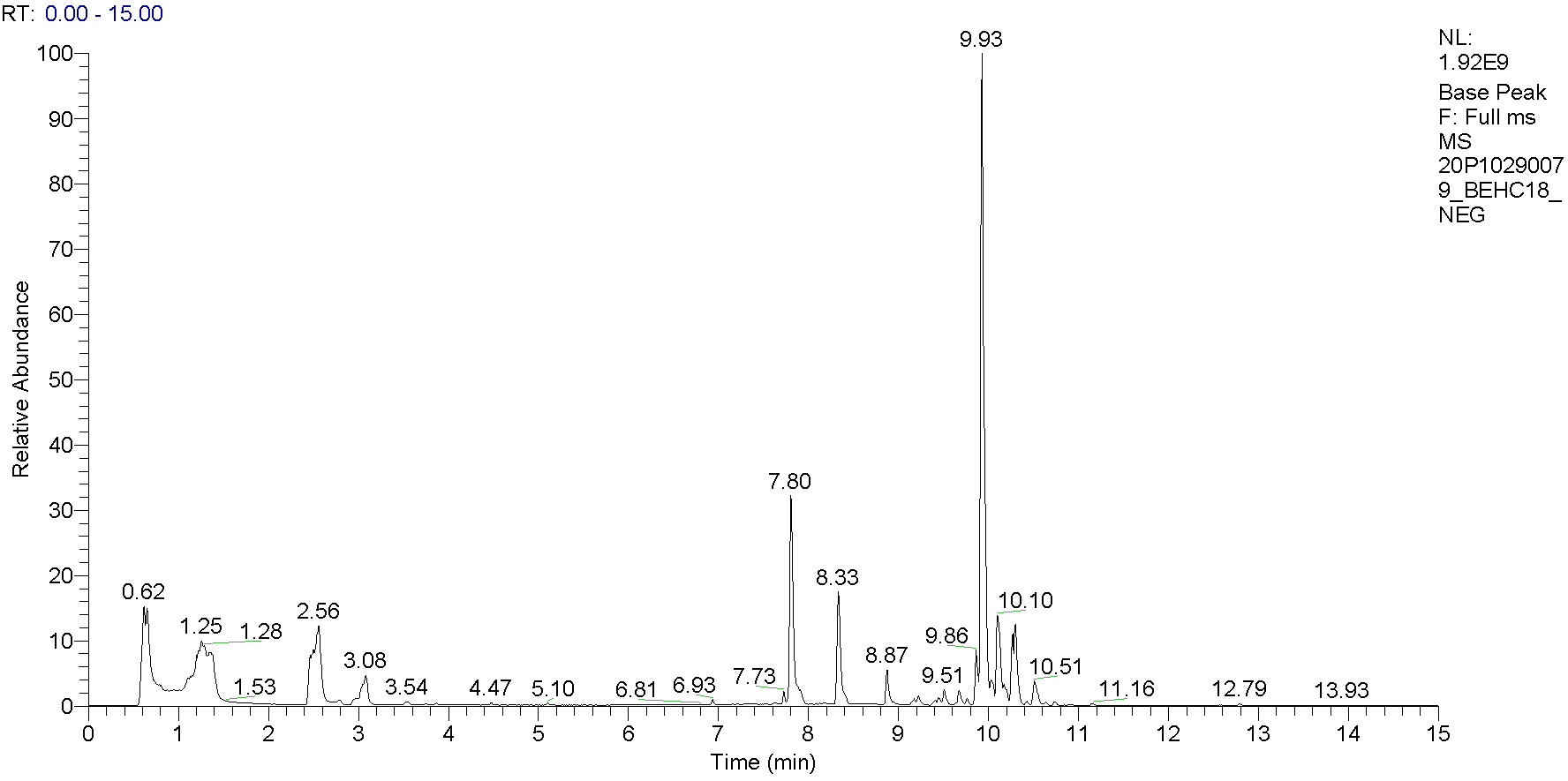


TD66-Du_BPC_pos


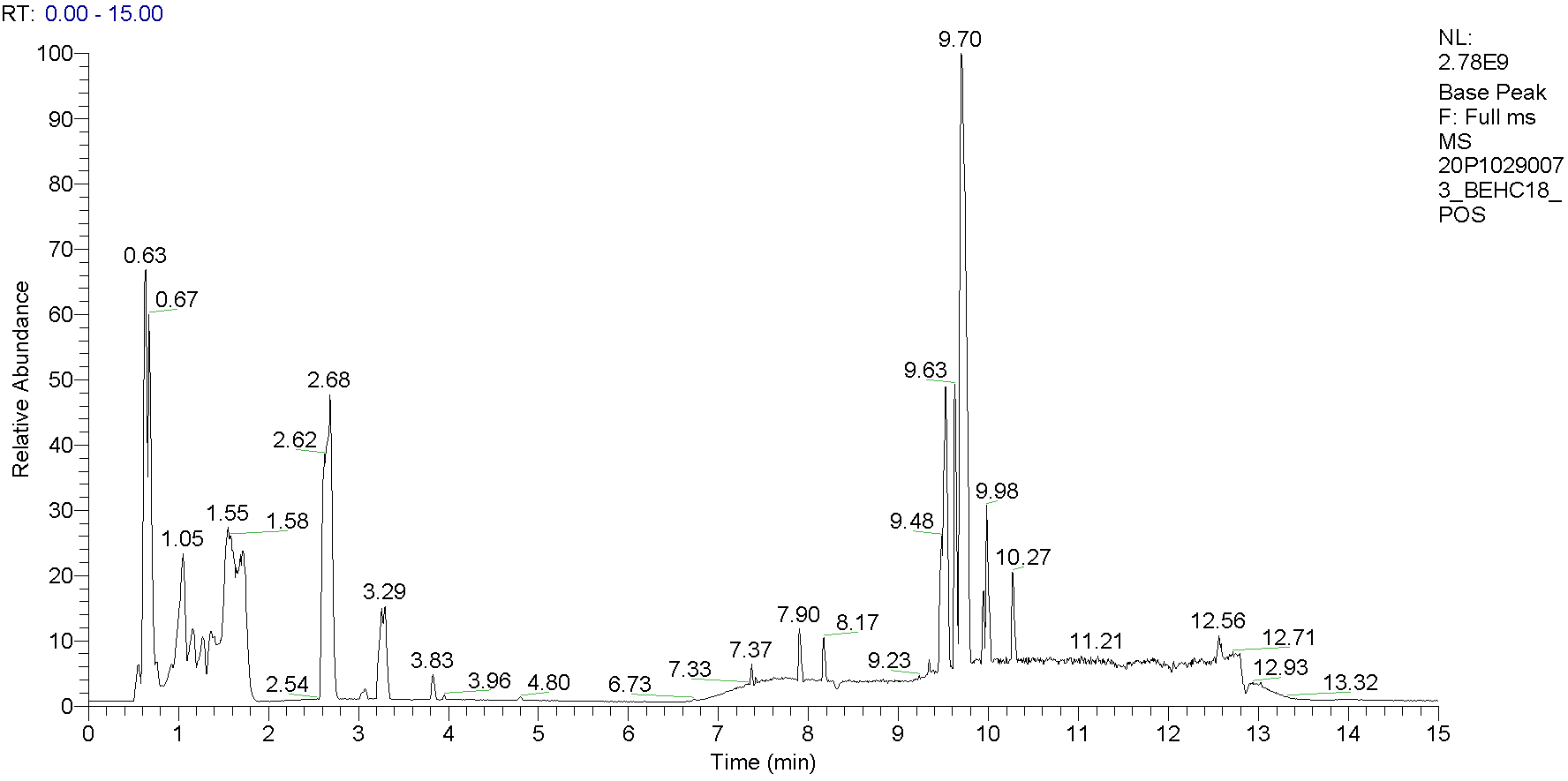


TD90-Du_BPC_neg


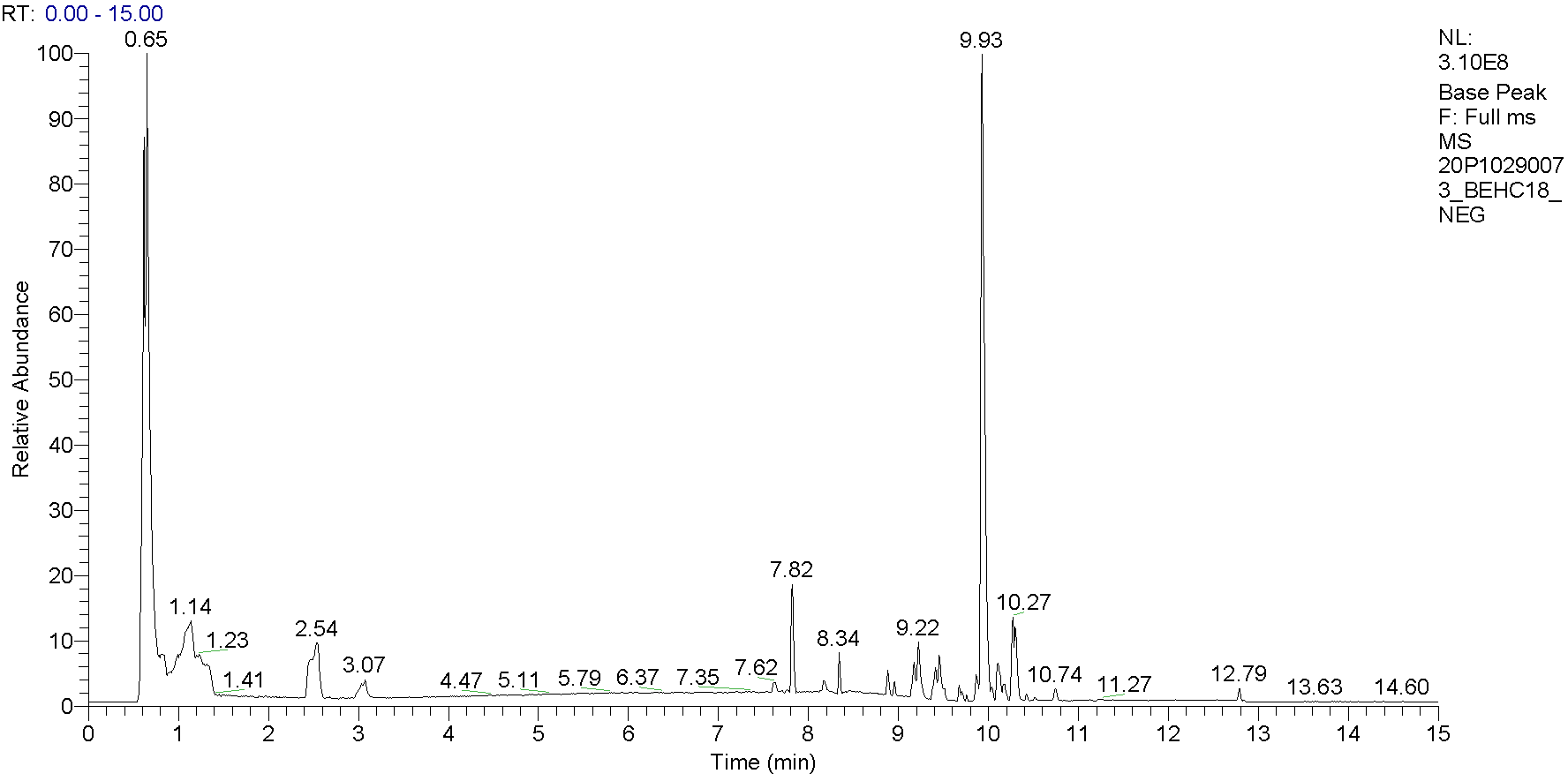


TD90-Du_BPC_pos


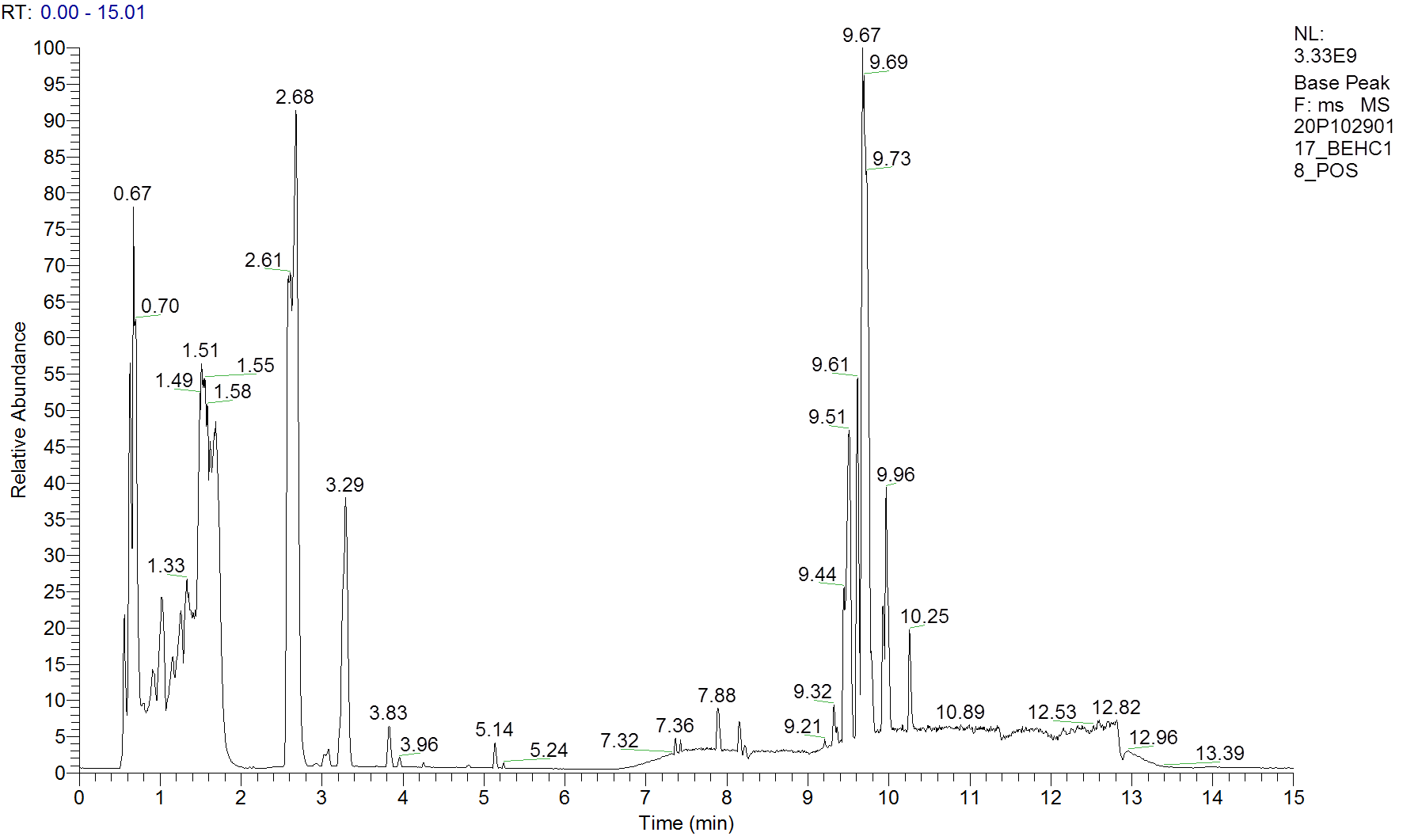


TD114-Du_BPC_neg


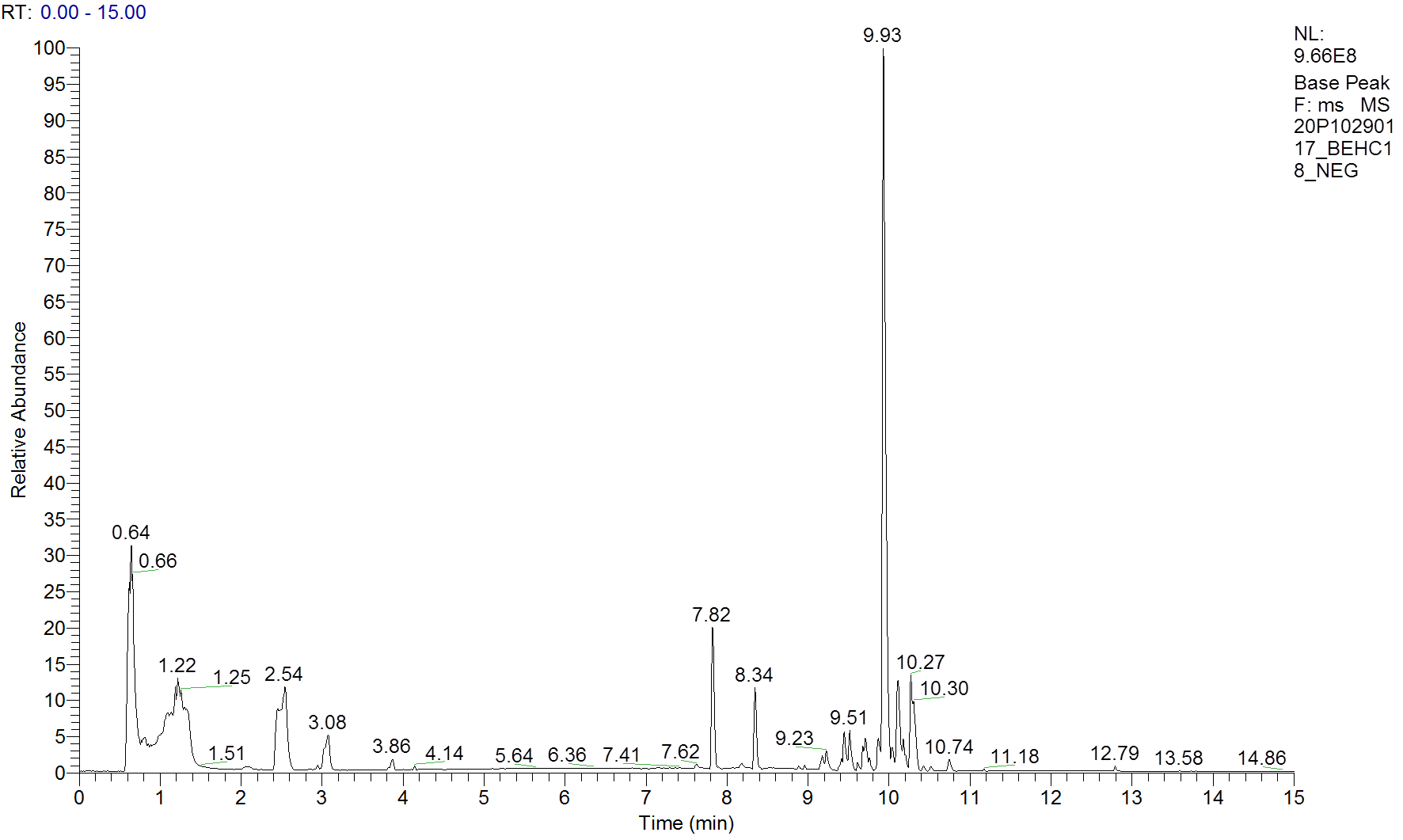


TD114-Du_BPC_pos


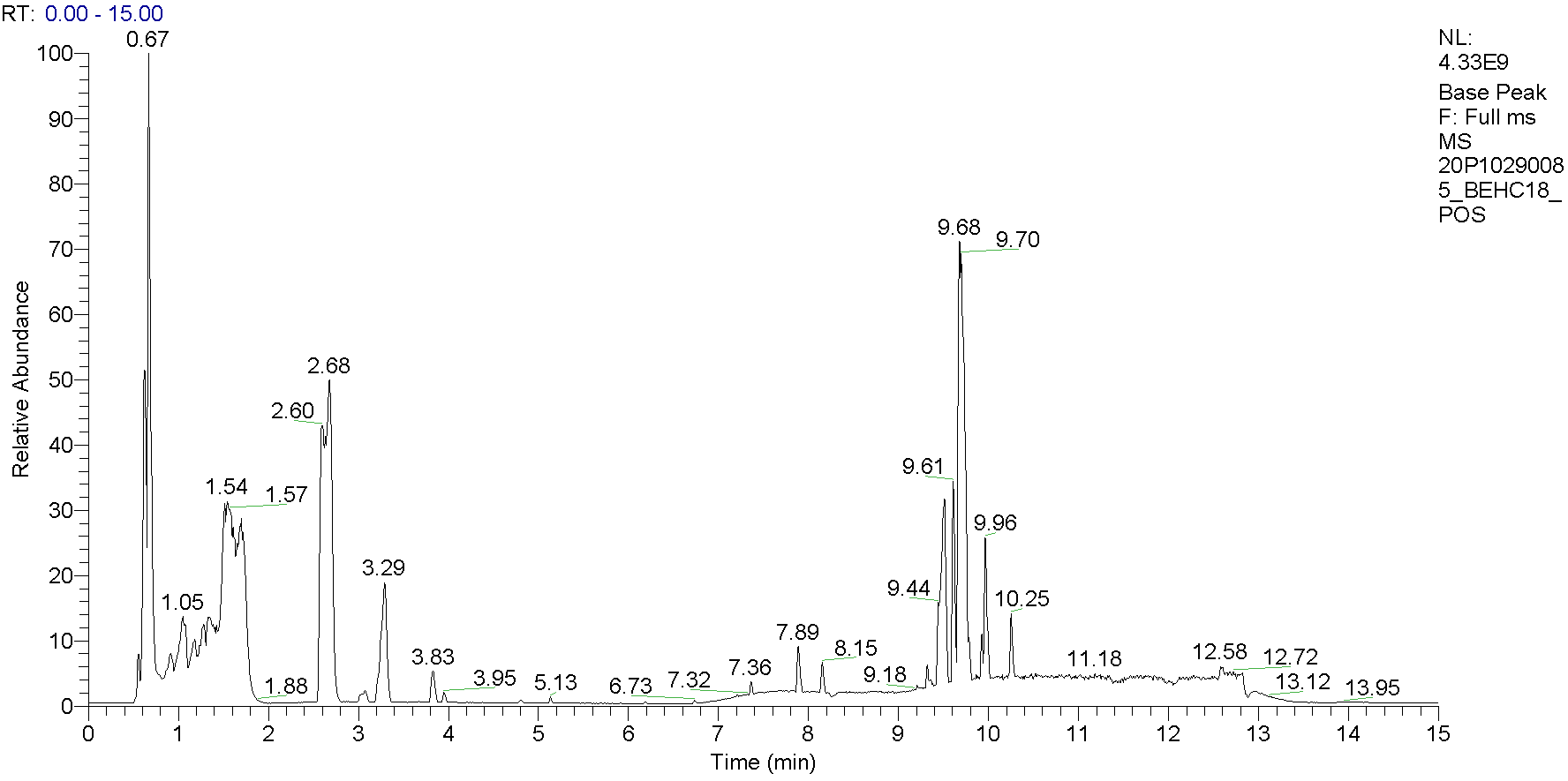


CK-I_BPC_neg


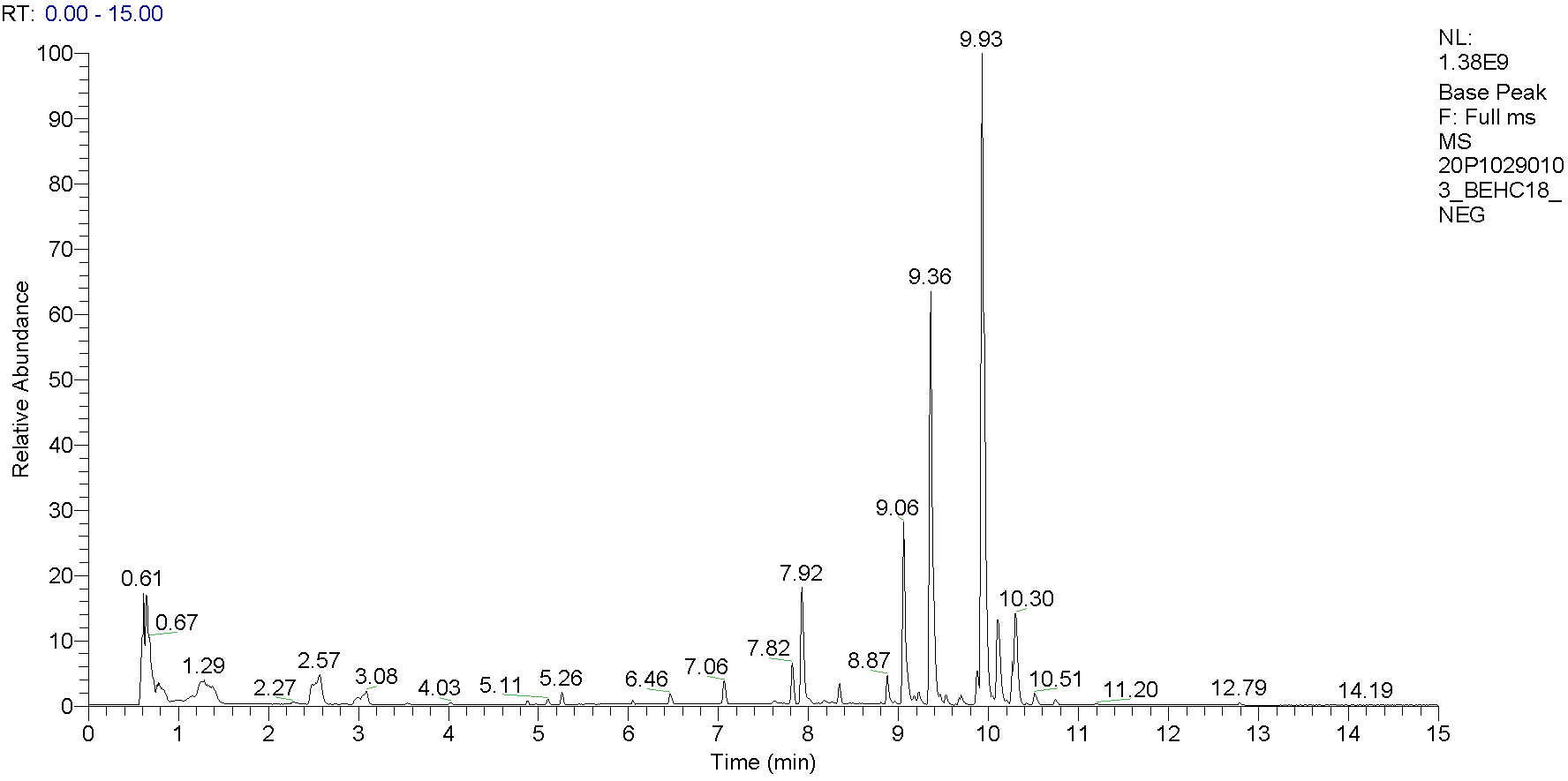


CK-I_BPC_pos


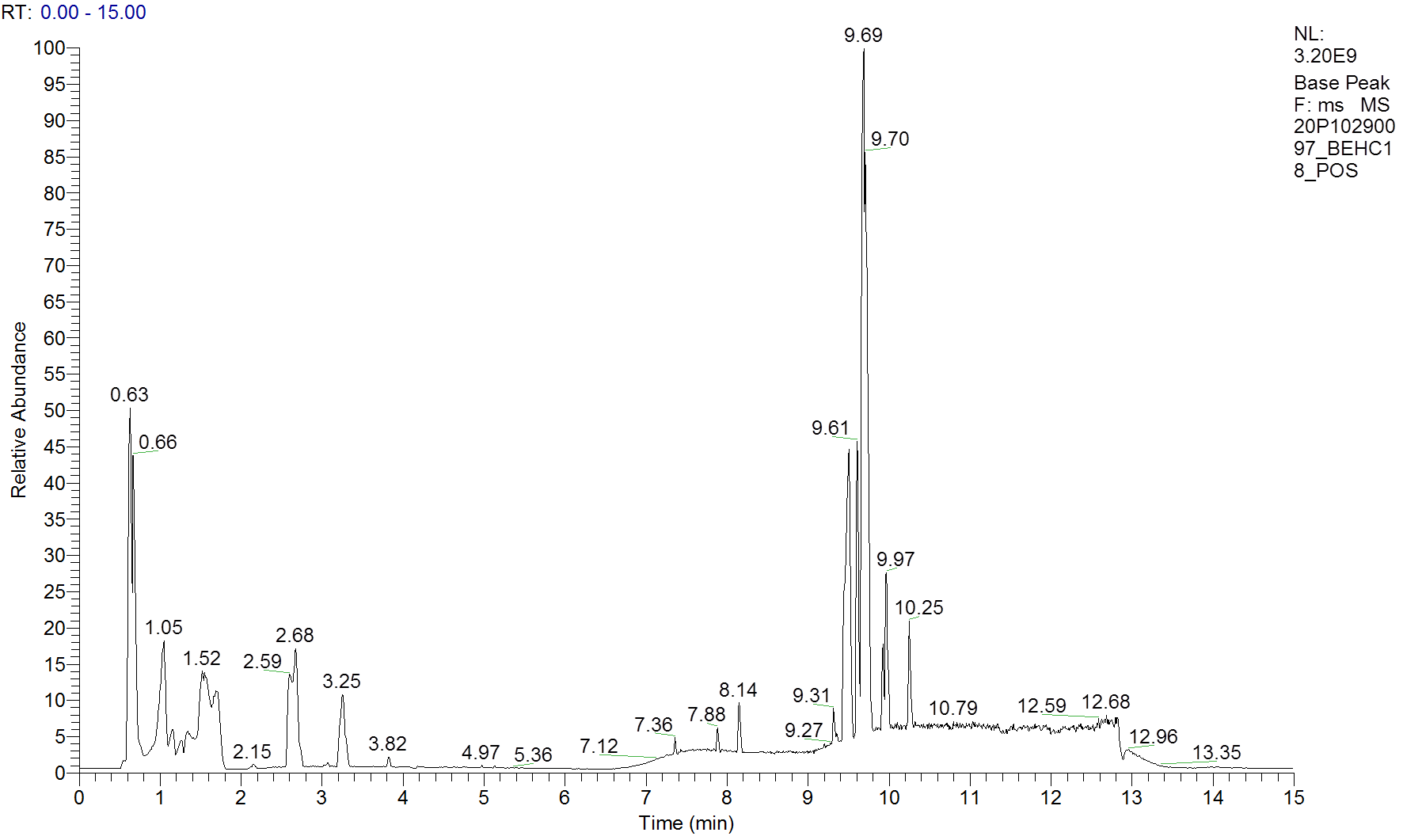


TD66-I_BPC_neg


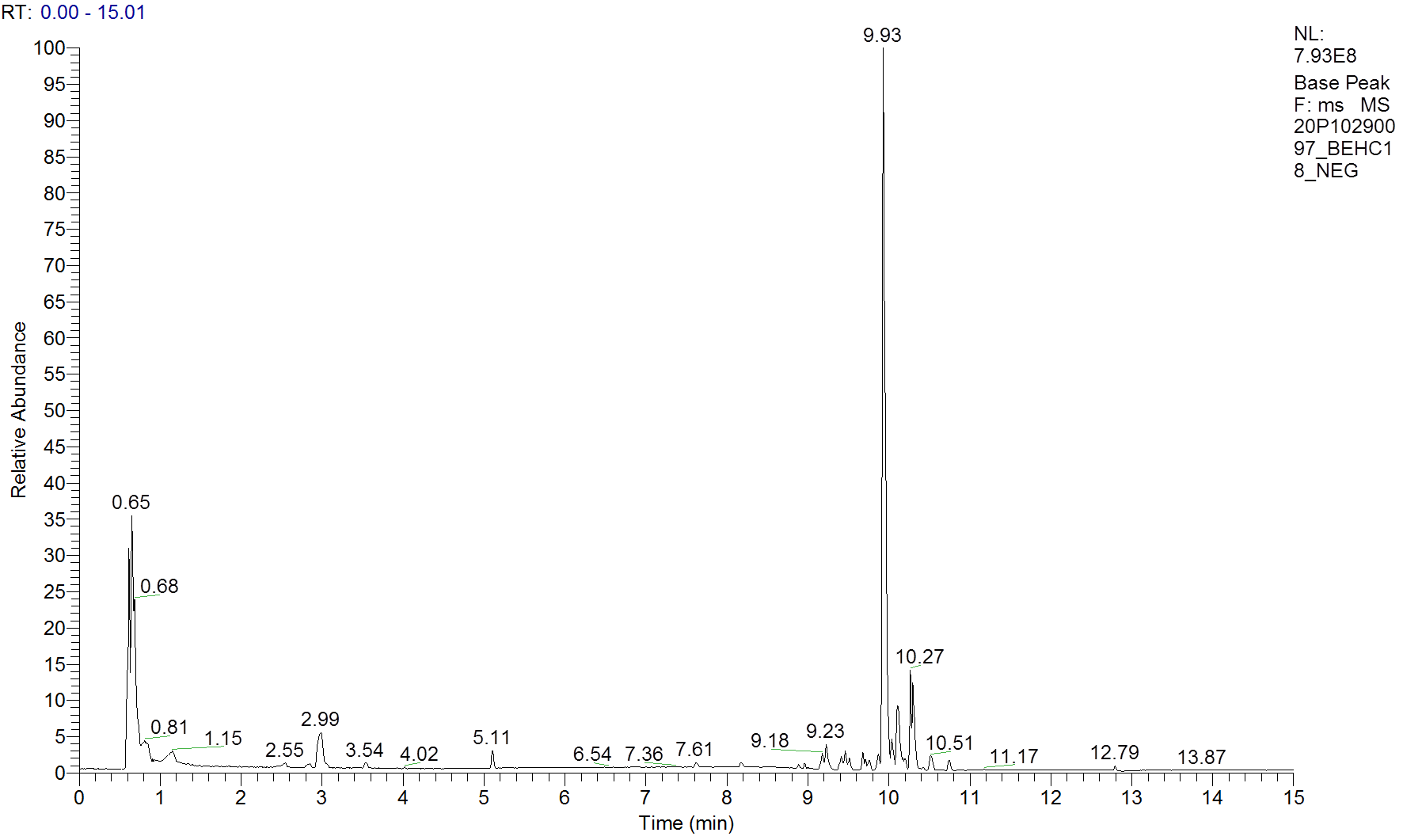


TD66-I_BPC_pos


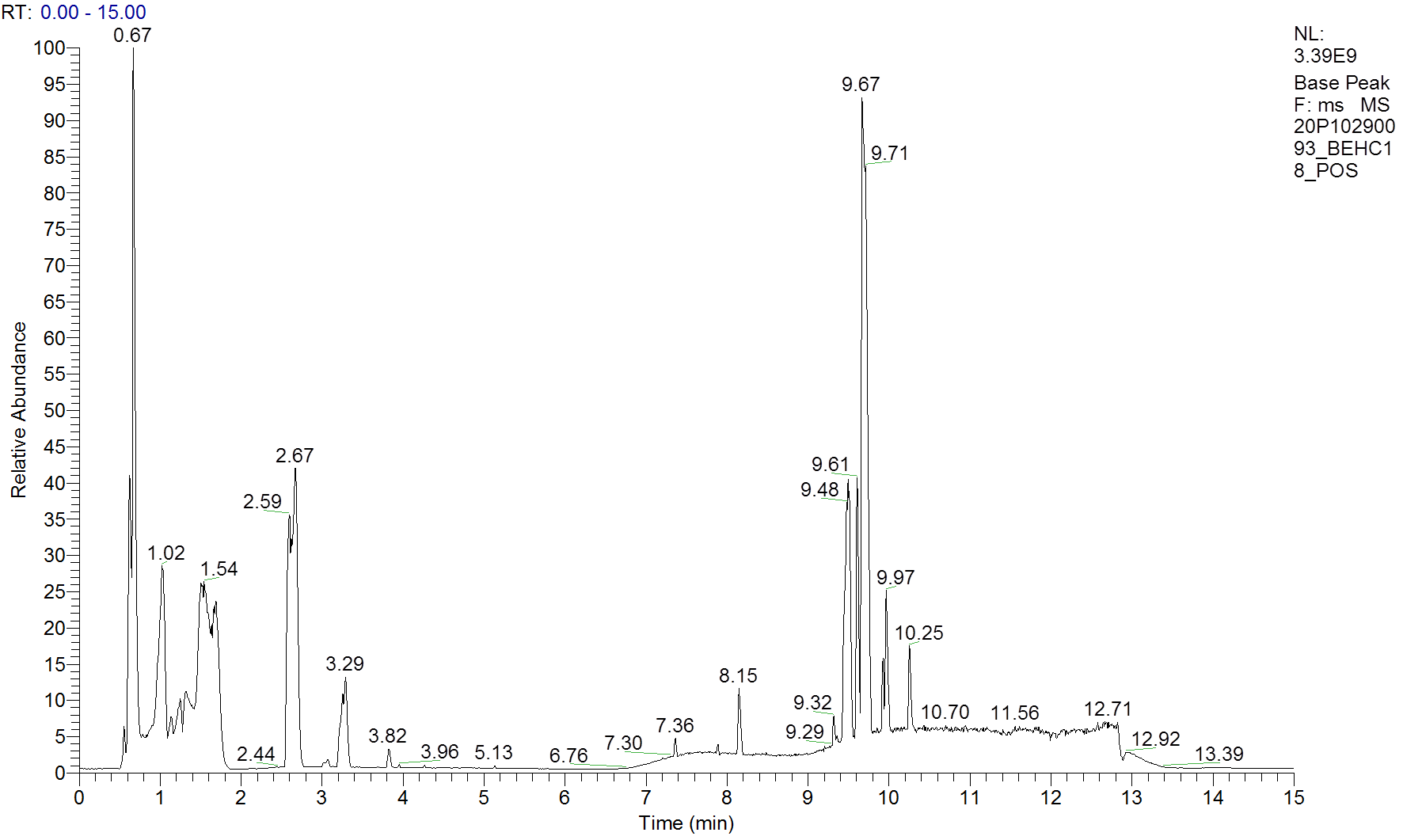


TD90-I_BPC_neg


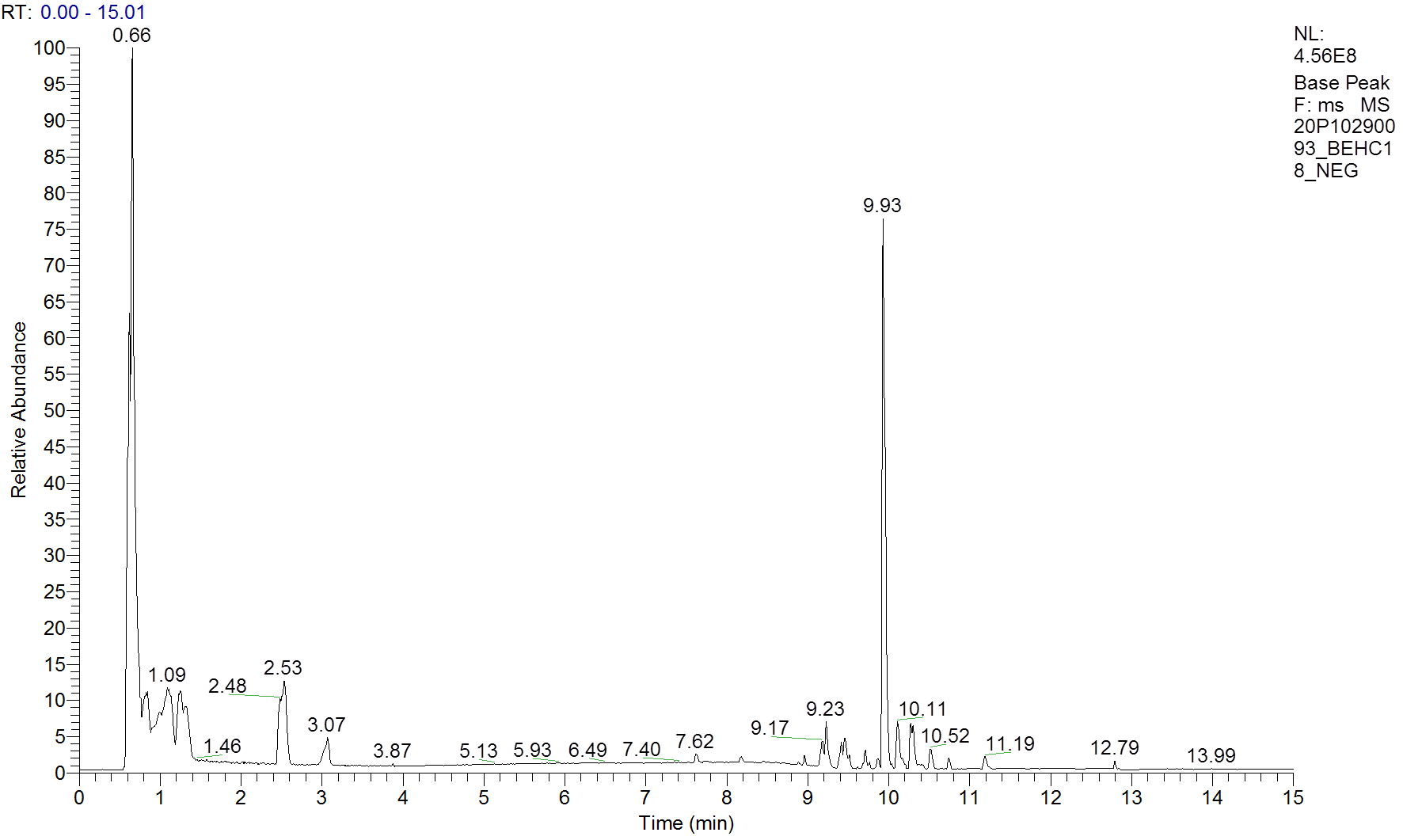


TD90-I_BPC_pos


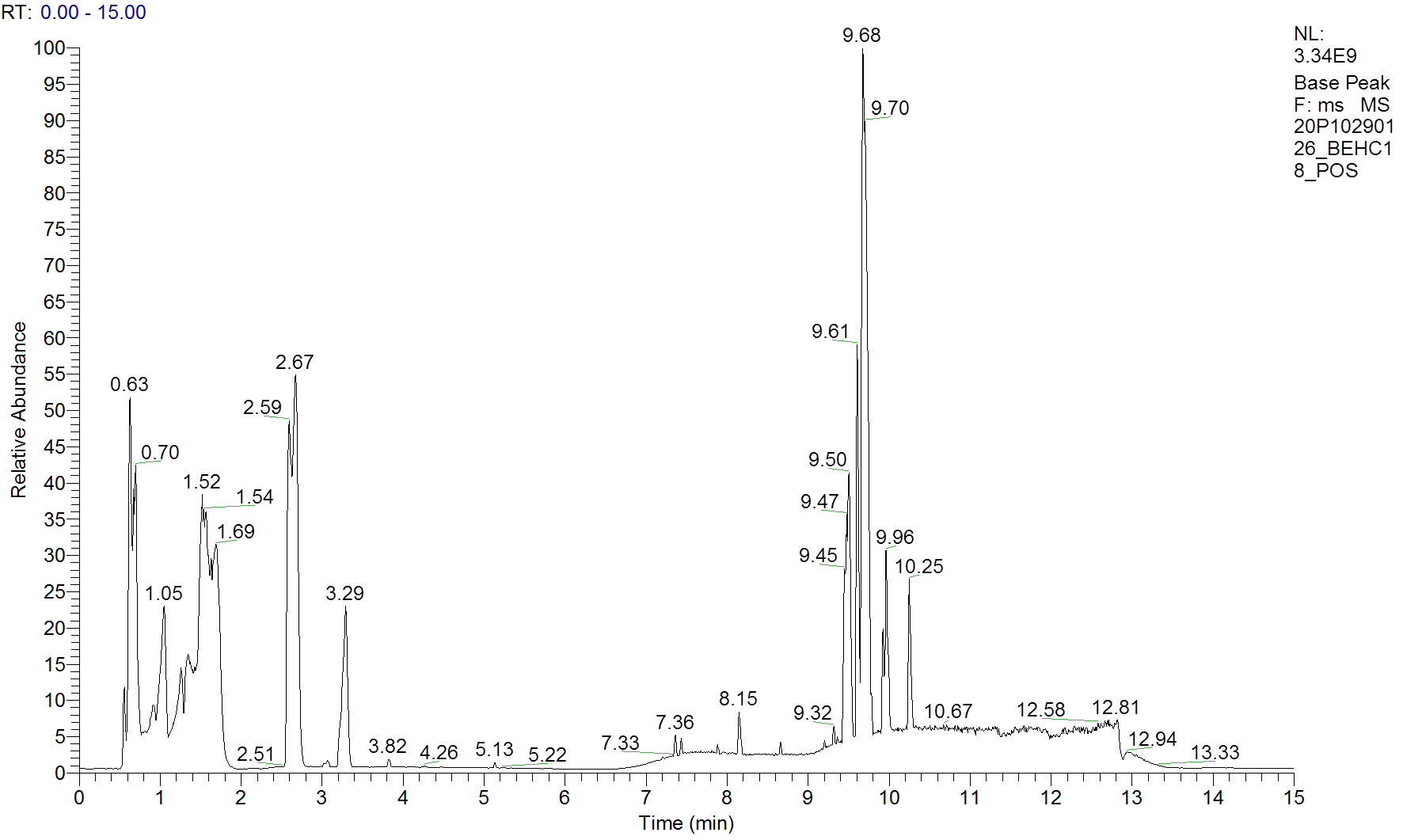


TD114-I_BPC_neg


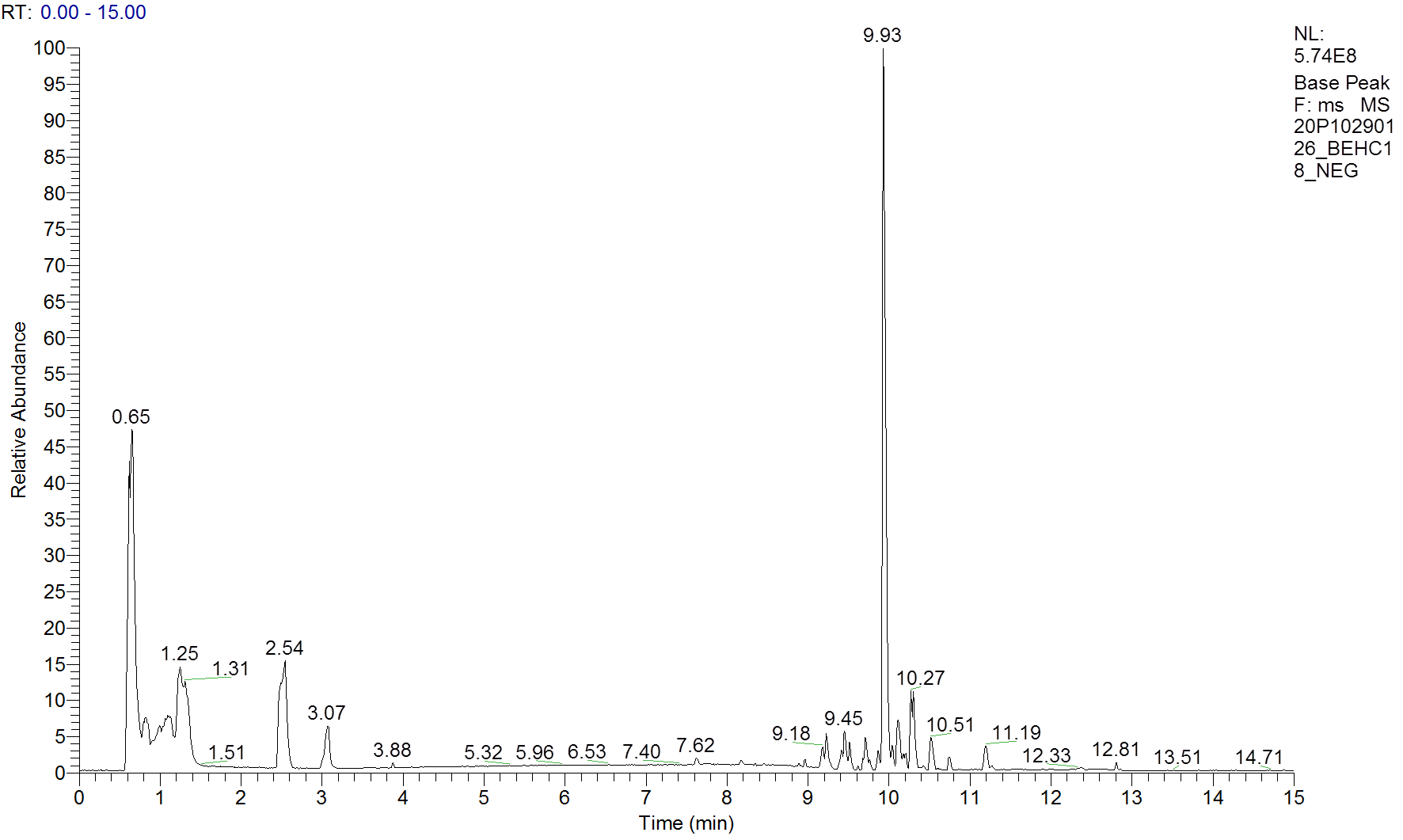


TD114-I_BPC_pos


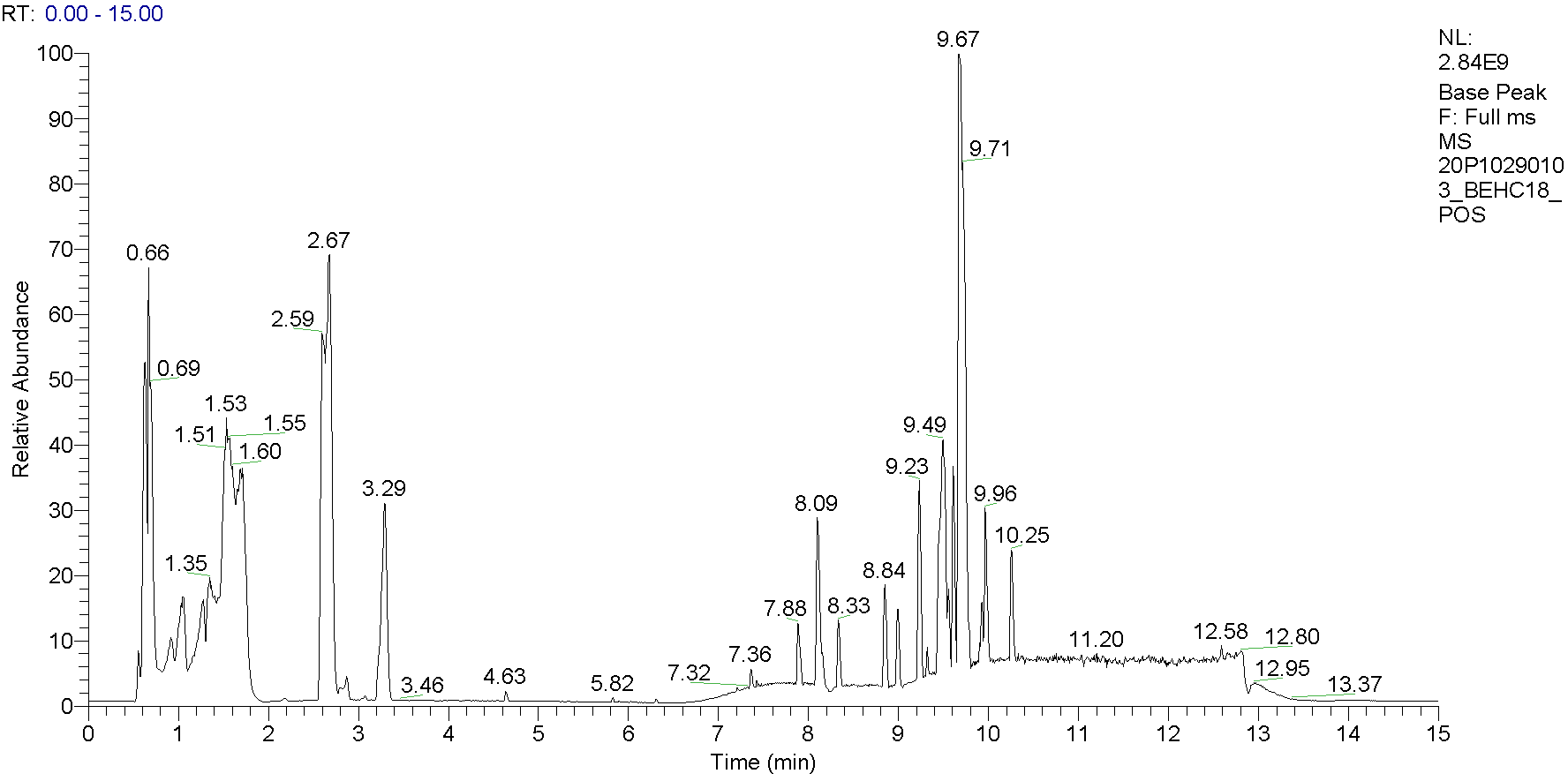

Supplement: Supplementary file 1 — Additional file 1. BPC chart. [file 12985_2023_2266_MOESM1_ESM.doc]
